# Supplementary figures and images for: SOX10 mediates glioblastoma cell-state plasticity
Source: EMBO Rep. 2024 Sep 16;25(11):24. doi: 10.1038/s44319-024-00258-8 (PMC11549307; doi:10.1038/s44319-024-00258-8)

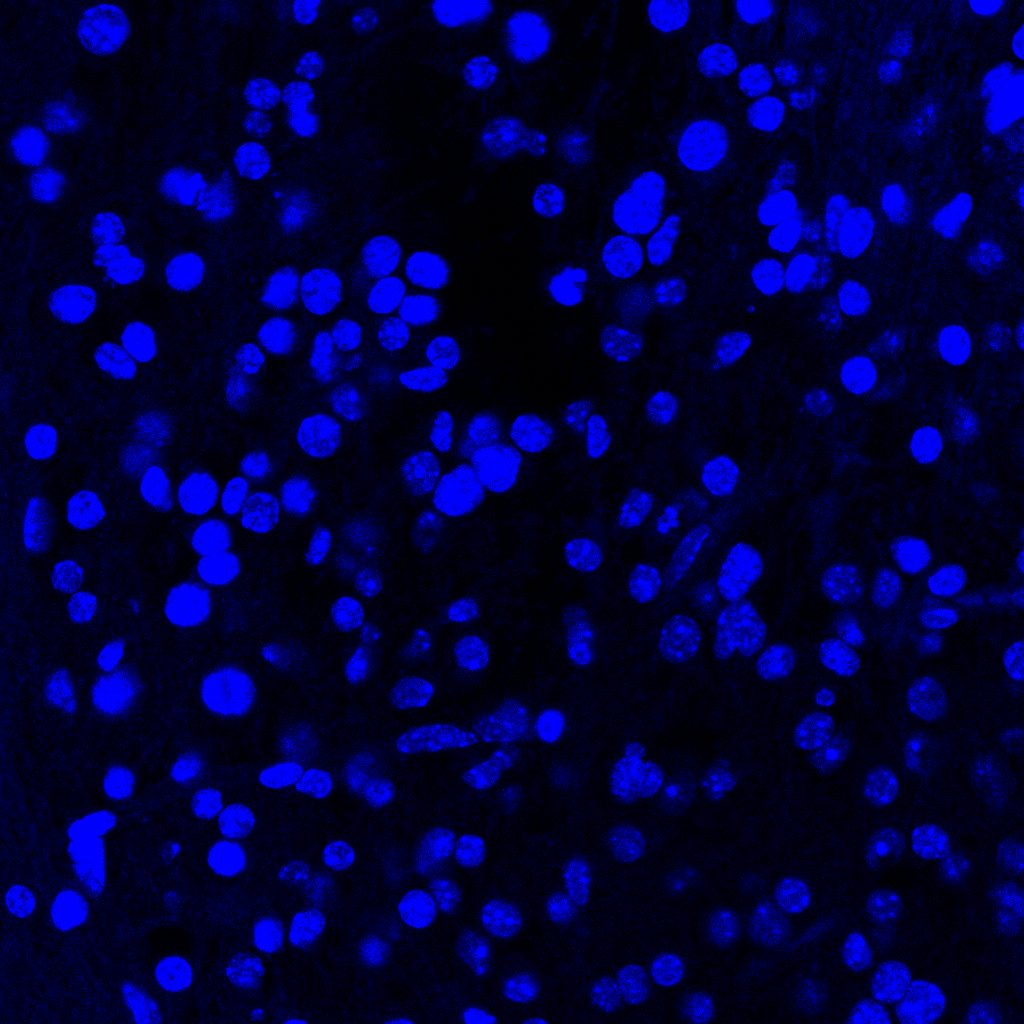

Supplement: Supplementary file 3 — Source data Fig. 1 [file 44319_2024_258_MOESM3_ESM.zip › Figure1/Fig1E/Sox10_GFP_IF/Ctrl_Sox10_DAPI.jpg]

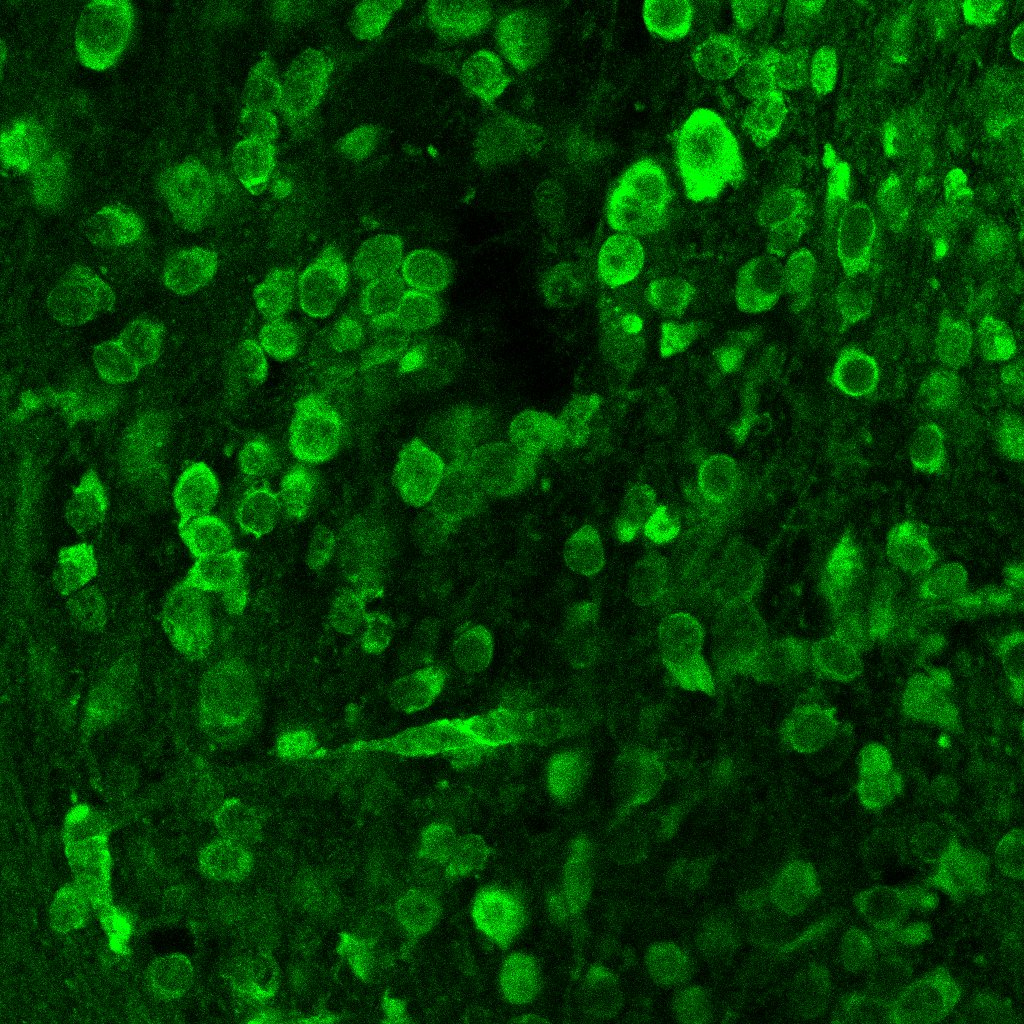

Supplement: Supplementary file 3 — Source data Fig. 1 [file 44319_2024_258_MOESM3_ESM.zip › Figure1/Fig1E/Sox10_GFP_IF/Ctrl_Sox10_GFP.jpg]

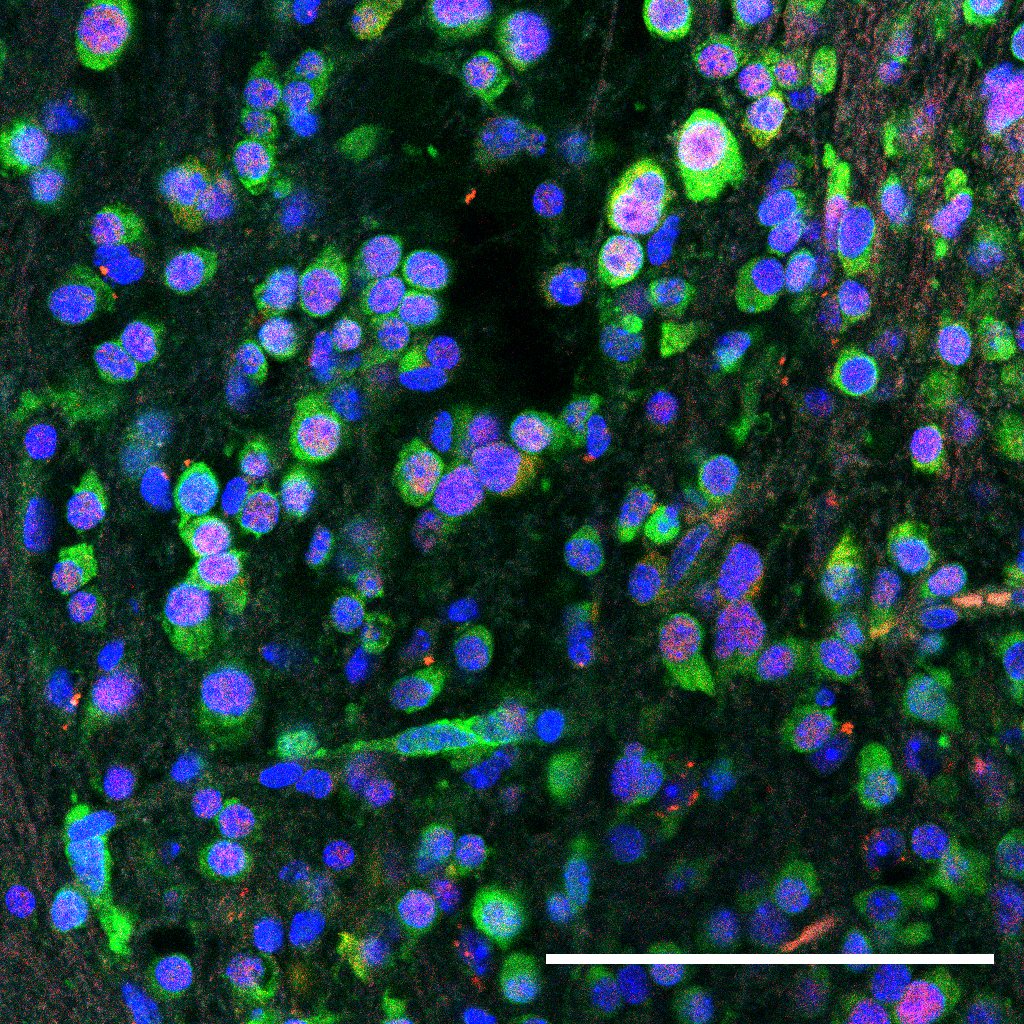

Supplement: Supplementary file 3 — Source data Fig. 1 [file 44319_2024_258_MOESM3_ESM.zip › Figure1/Fig1E/Sox10_GFP_IF/Ctrl_Sox10_merge.jpg]

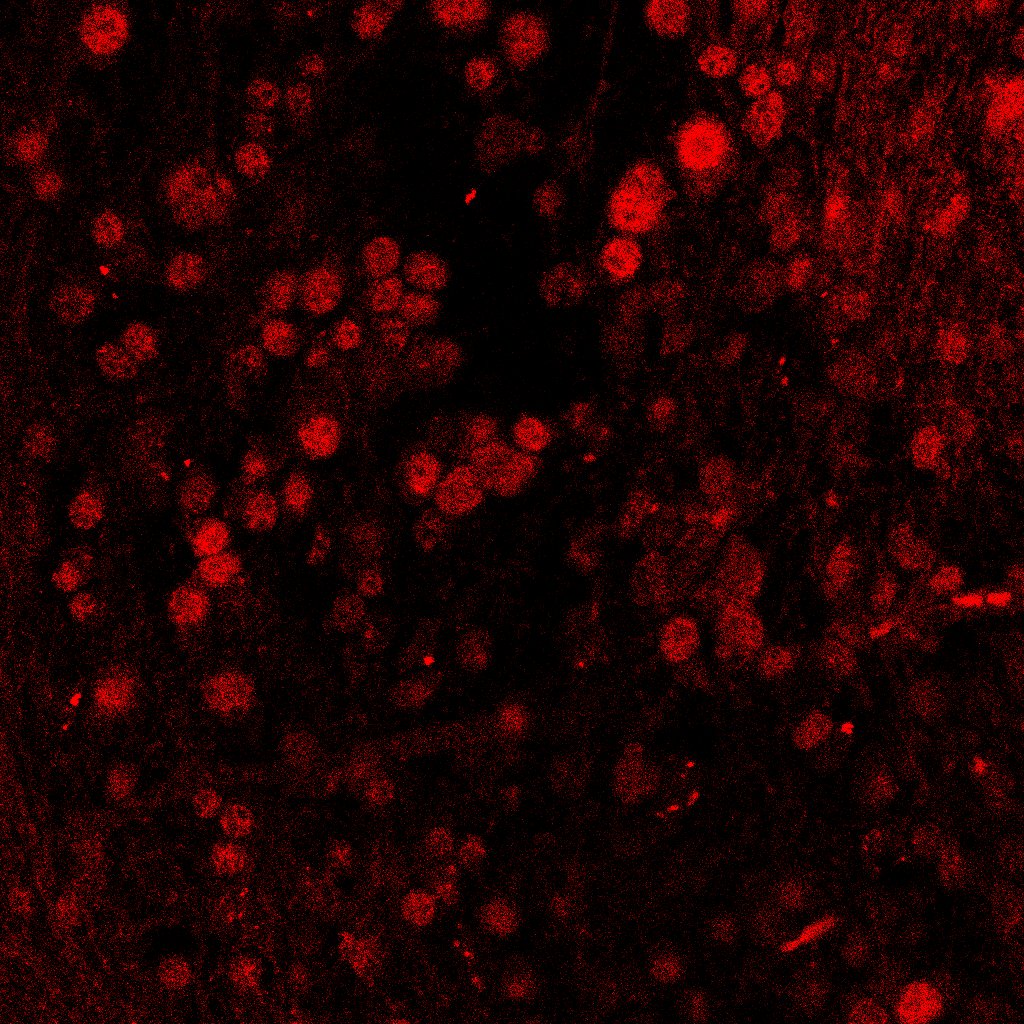

Supplement: Supplementary file 3 — Source data Fig. 1 [file 44319_2024_258_MOESM3_ESM.zip › Figure1/Fig1E/Sox10_GFP_IF/Ctrl_Sox10_Sox10.jpg]

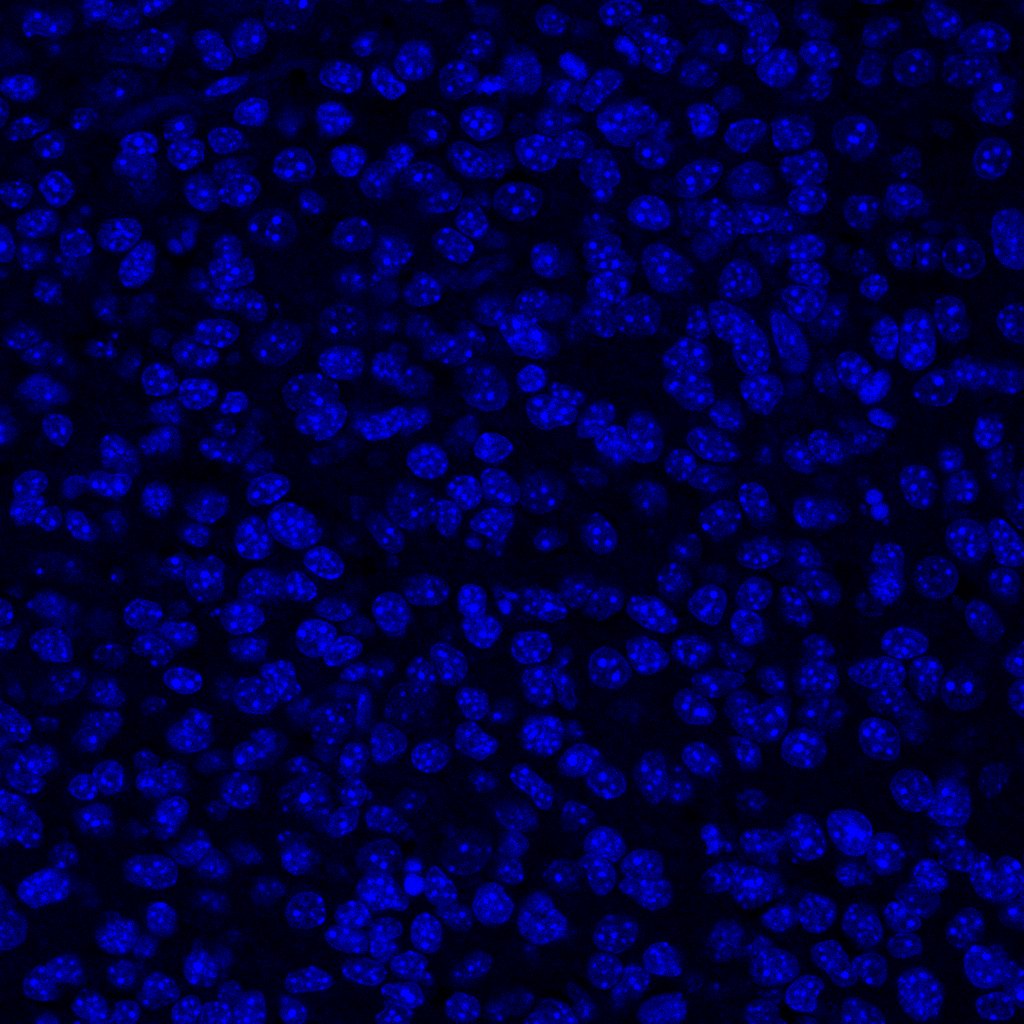

Supplement: Supplementary file 3 — Source data Fig. 1 [file 44319_2024_258_MOESM3_ESM.zip › Figure1/Fig1E/Sox10_GFP_IF/KD_Sox10_DAPI.jpg]

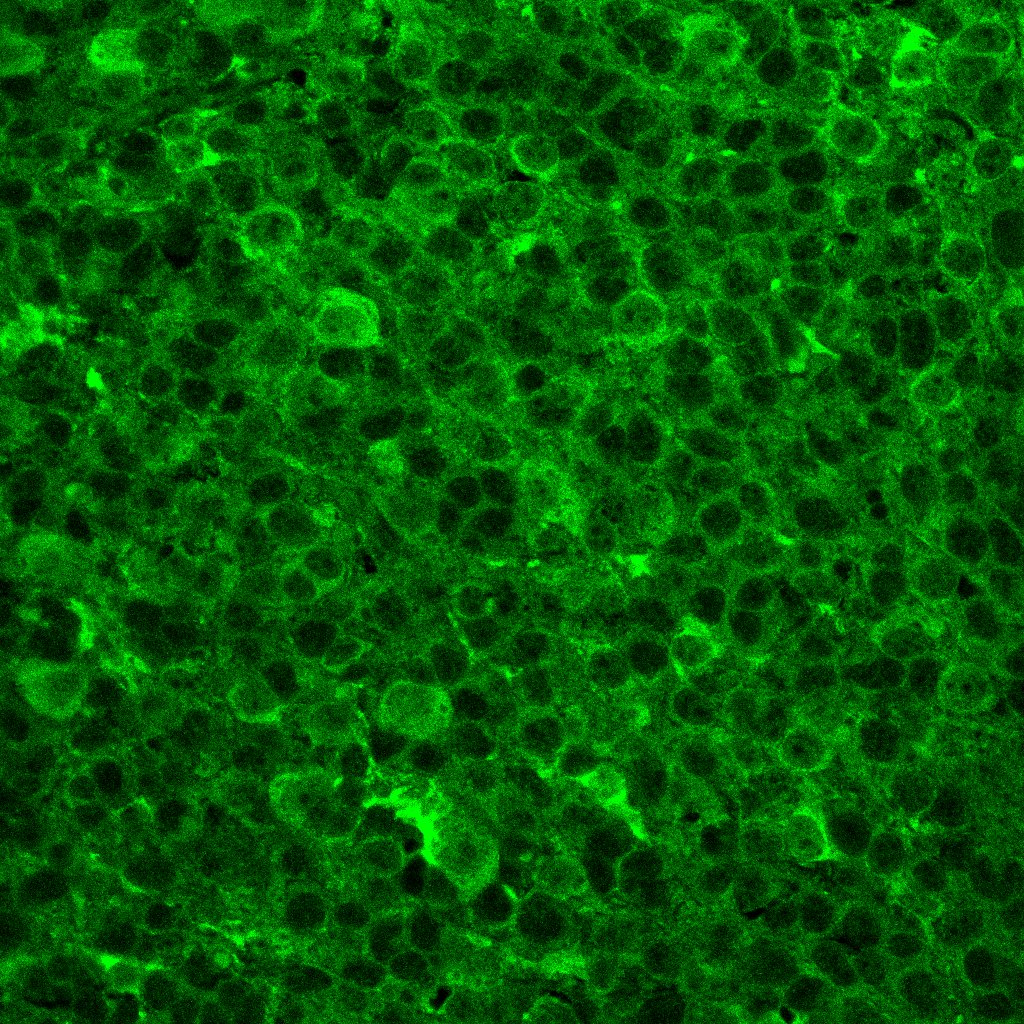

Supplement: Supplementary file 3 — Source data Fig. 1 [file 44319_2024_258_MOESM3_ESM.zip › Figure1/Fig1E/Sox10_GFP_IF/KD_Sox10_GFP.jpg]

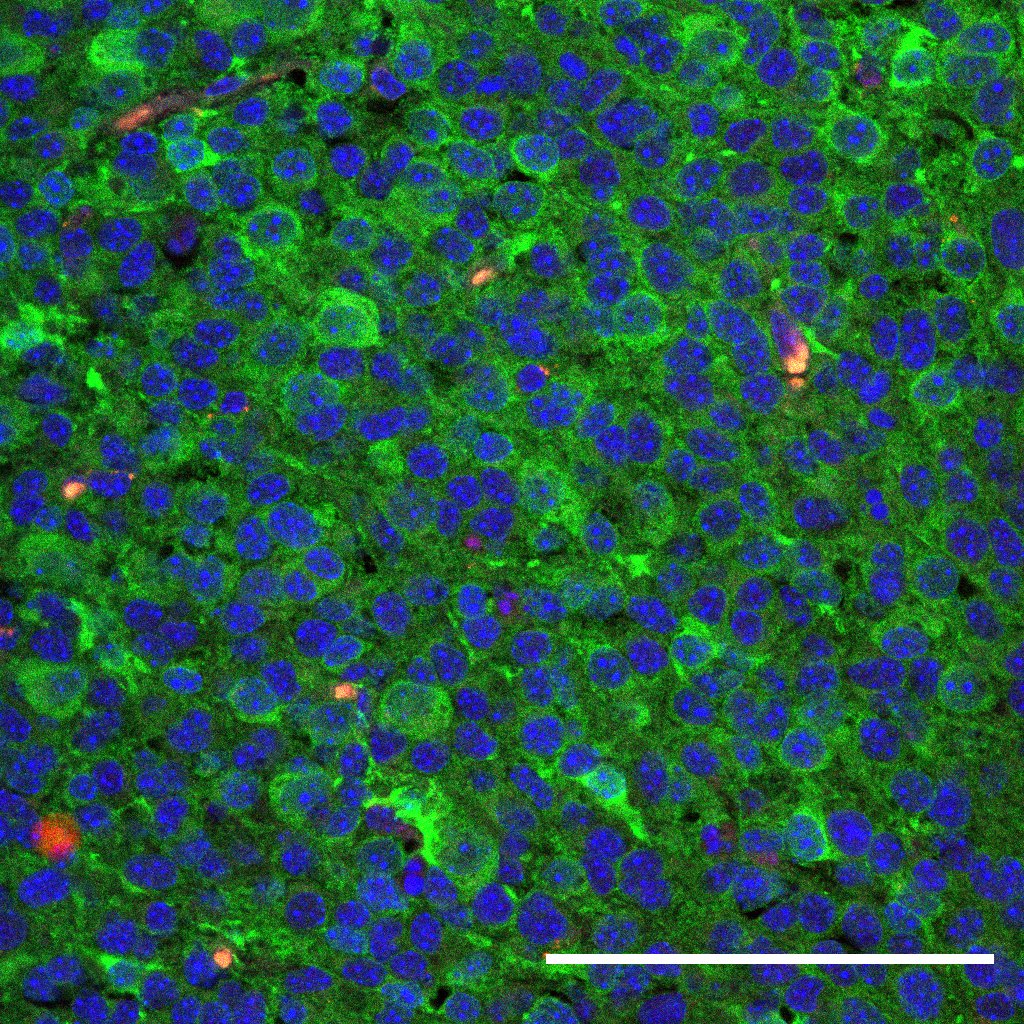

Supplement: Supplementary file 3 — Source data Fig. 1 [file 44319_2024_258_MOESM3_ESM.zip › Figure1/Fig1E/Sox10_GFP_IF/KD_Sox10_merge.jpg]

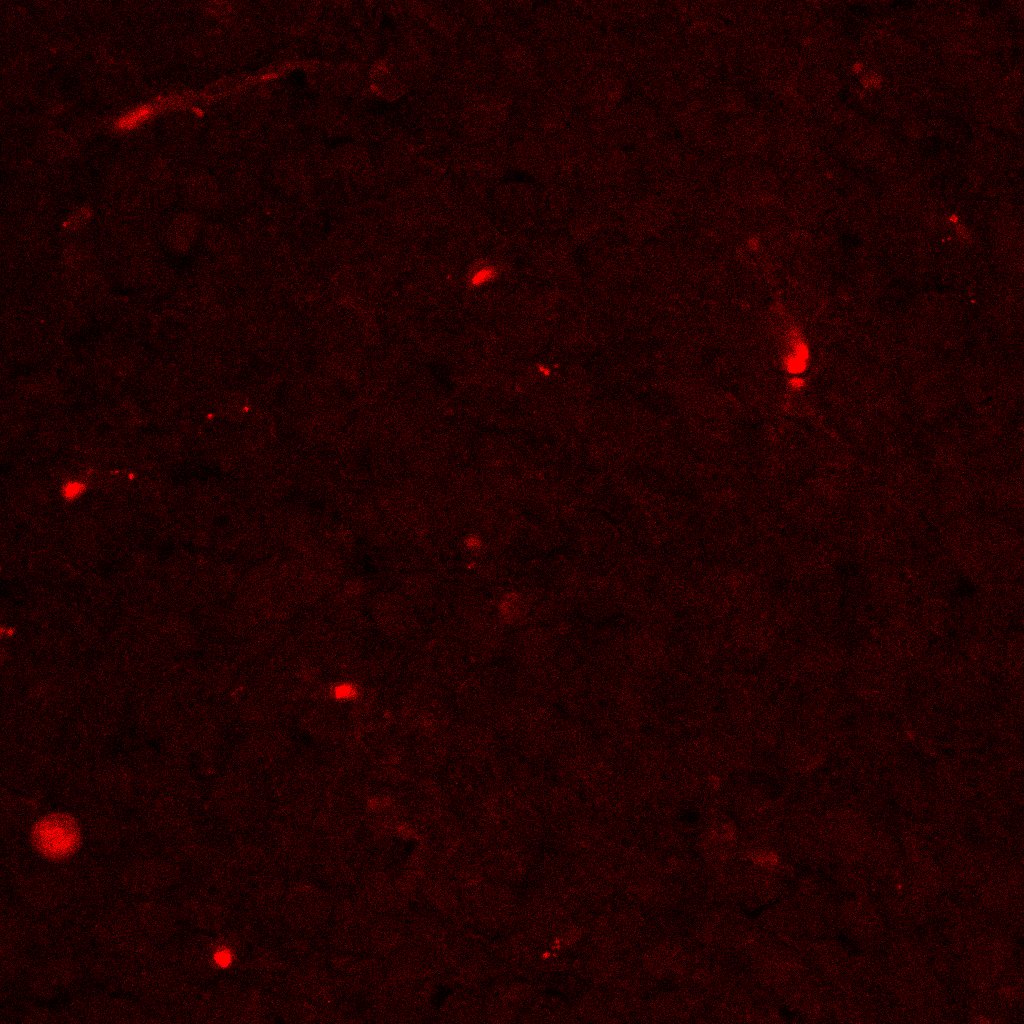

Supplement: Supplementary file 3 — Source data Fig. 1 [file 44319_2024_258_MOESM3_ESM.zip › Figure1/Fig1E/Sox10_GFP_IF/KD_Sox10_Sox10.jpg]

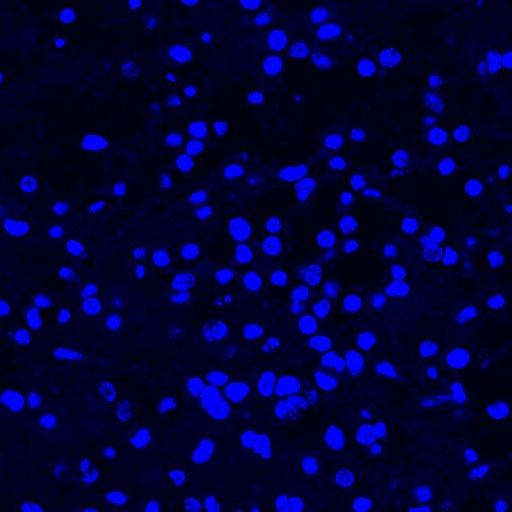

Supplement: Supplementary file 3 — Source data Fig. 1 [file 44319_2024_258_MOESM3_ESM.zip › Figure1/Fig1E/Sox9_GFP_IF/Ctrl_Sox9_DAPI.jpg]

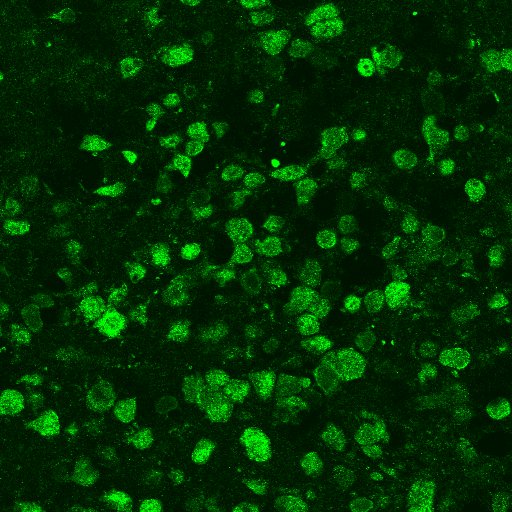

Supplement: Supplementary file 3 — Source data Fig. 1 [file 44319_2024_258_MOESM3_ESM.zip › Figure1/Fig1E/Sox9_GFP_IF/Ctrl_Sox9_GFP.jpg]

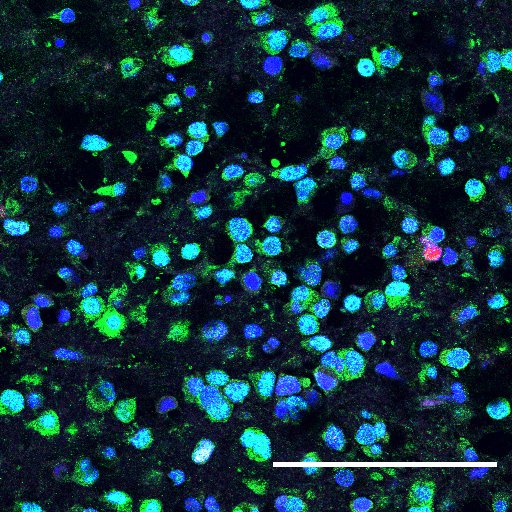

Supplement: Supplementary file 3 — Source data Fig. 1 [file 44319_2024_258_MOESM3_ESM.zip › Figure1/Fig1E/Sox9_GFP_IF/Ctrl_Sox9_Merge.jpg]

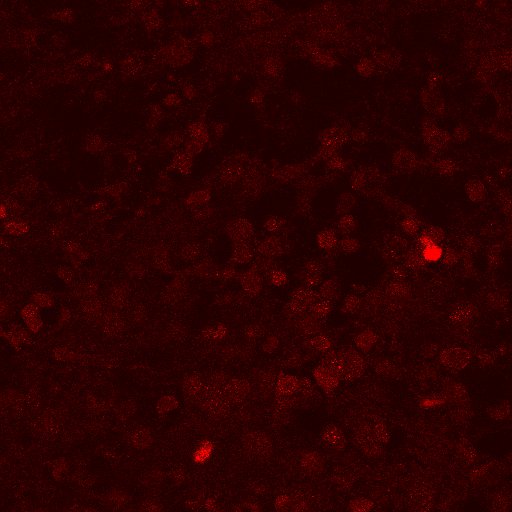

Supplement: Supplementary file 3 — Source data Fig. 1 [file 44319_2024_258_MOESM3_ESM.zip › Figure1/Fig1E/Sox9_GFP_IF/Ctrl_Sox9_Sox9.jpg]

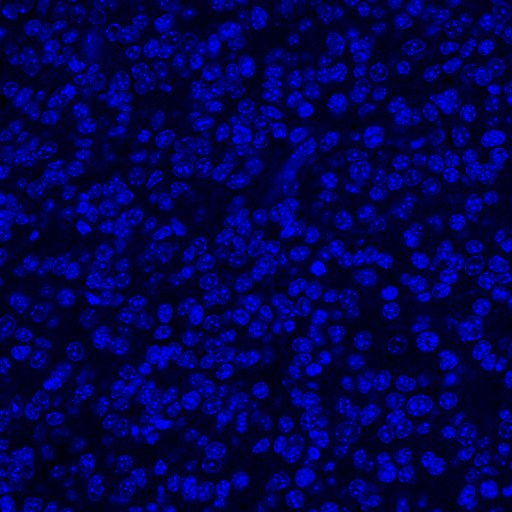

Supplement: Supplementary file 3 — Source data Fig. 1 [file 44319_2024_258_MOESM3_ESM.zip › Figure1/Fig1E/Sox9_GFP_IF/KD_Sox9_DAPI.jpg]

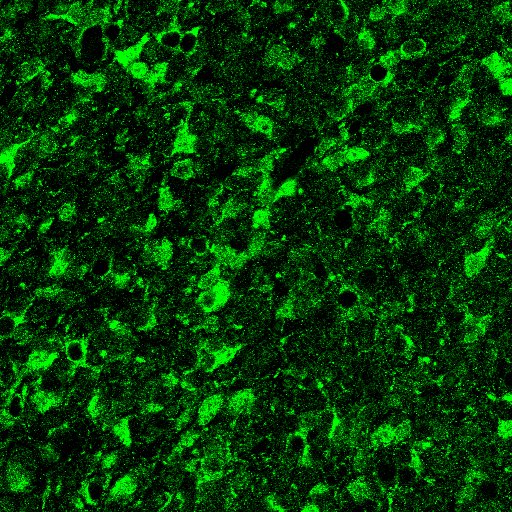

Supplement: Supplementary file 3 — Source data Fig. 1 [file 44319_2024_258_MOESM3_ESM.zip › Figure1/Fig1E/Sox9_GFP_IF/KD_Sox9_GFP.jpg]

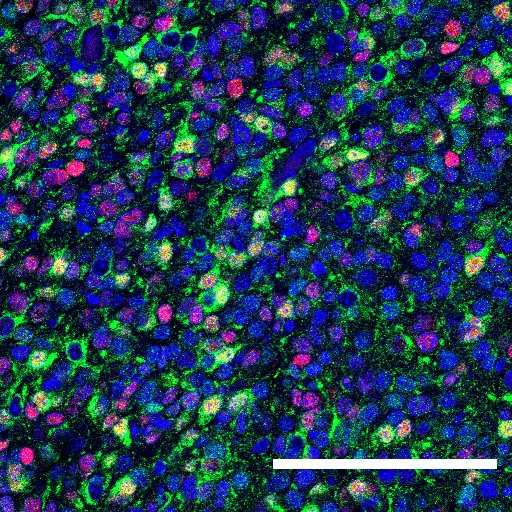

Supplement: Supplementary file 3 — Source data Fig. 1 [file 44319_2024_258_MOESM3_ESM.zip › Figure1/Fig1E/Sox9_GFP_IF/KD_Sox9_Merge.jpg]

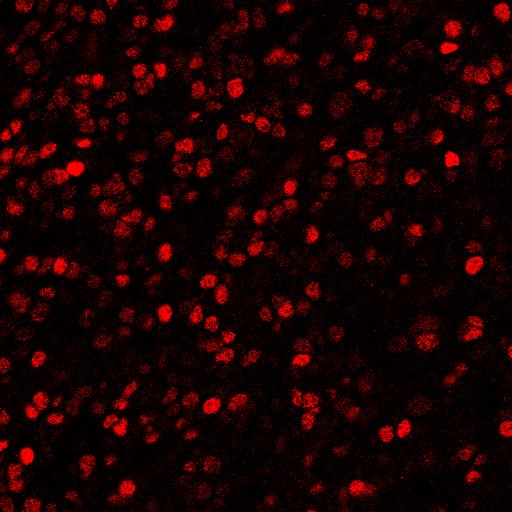

Supplement: Supplementary file 3 — Source data Fig. 1 [file 44319_2024_258_MOESM3_ESM.zip › Figure1/Fig1E/Sox9_GFP_IF/KD_Sox9_Sox9.jpg]

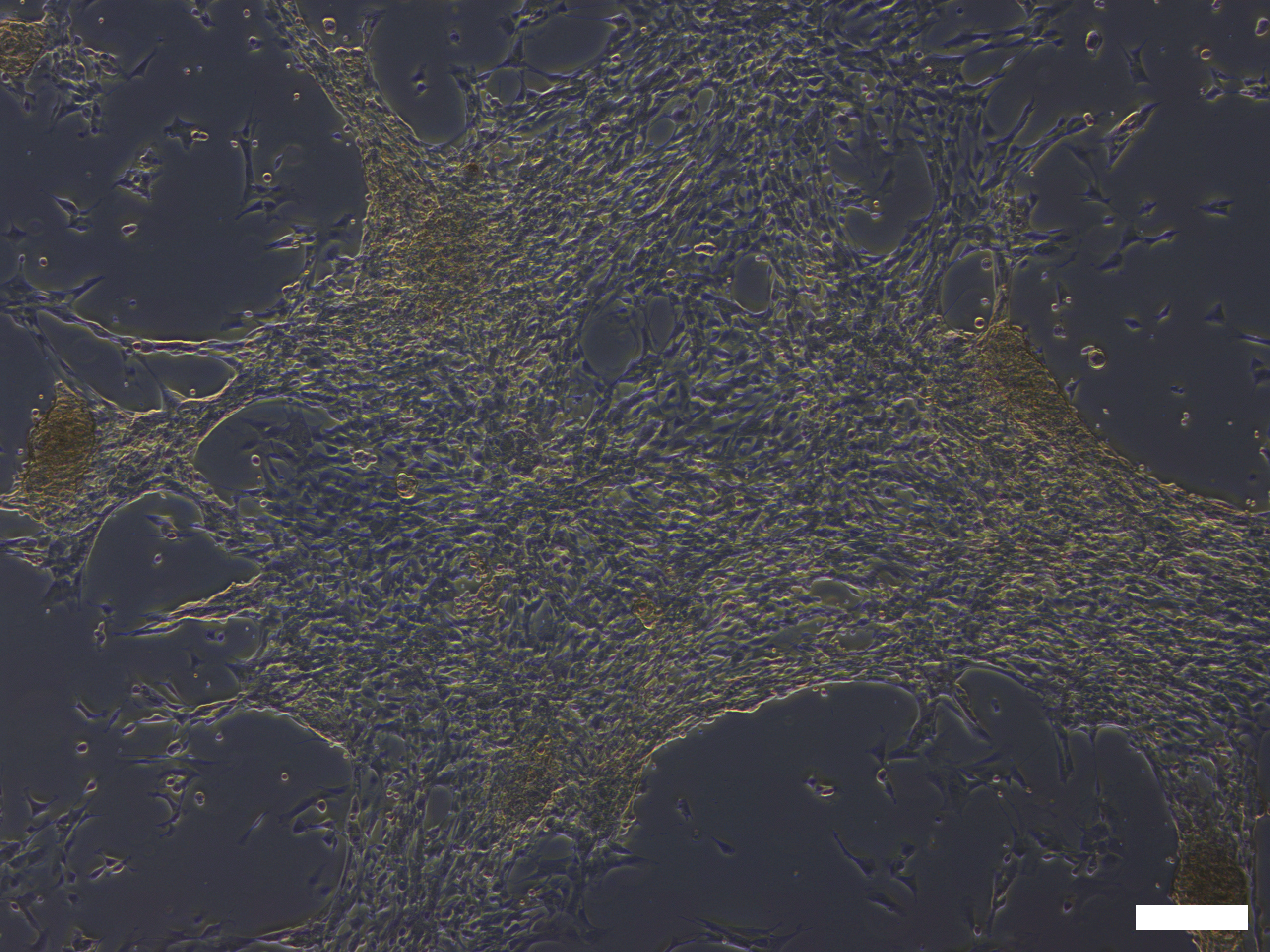

Supplement: Supplementary file 3 — Source data Fig. 1 [file 44319_2024_258_MOESM3_ESM.zip › Figure1/Fig1F/mGB1-Ctrl-Diff.tif]

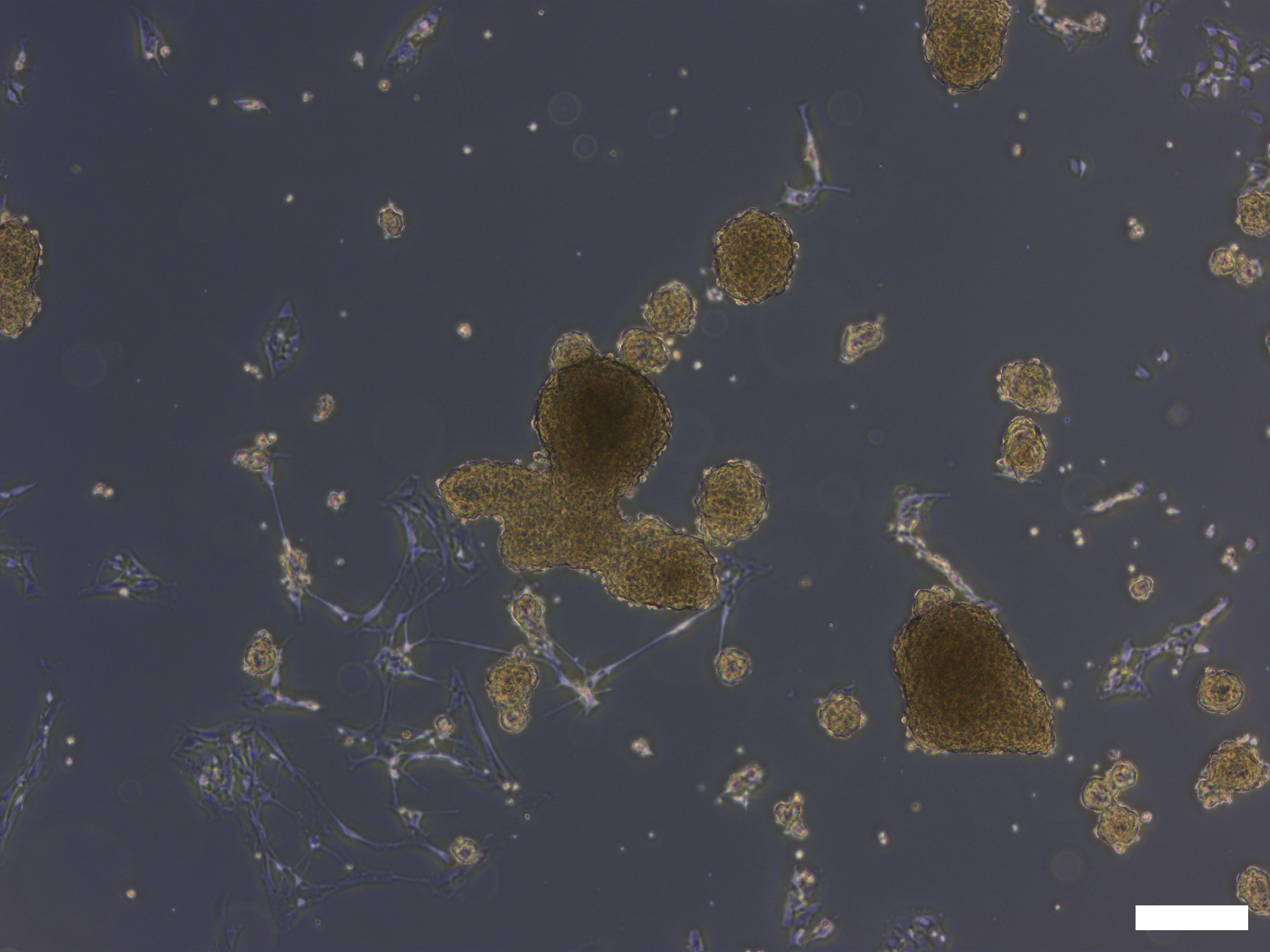

Supplement: Supplementary file 3 — Source data Fig. 1 [file 44319_2024_258_MOESM3_ESM.zip › Figure1/Fig1F/mGB1-KD1-Diff.tif]

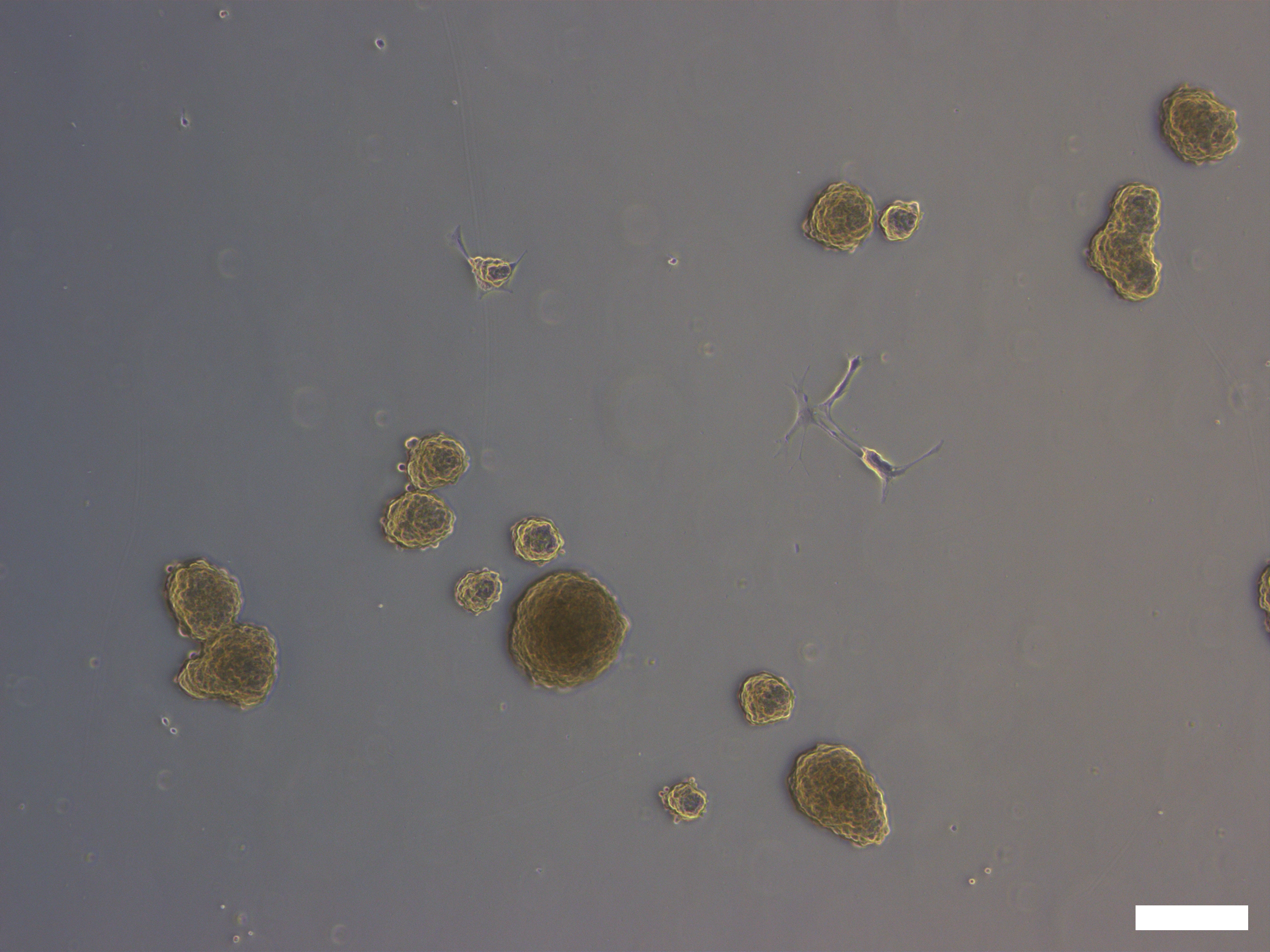

Supplement: Supplementary file 3 — Source data Fig. 1 [file 44319_2024_258_MOESM3_ESM.zip › Figure1/Fig1F/mGB1-KD2-Diff.tif]

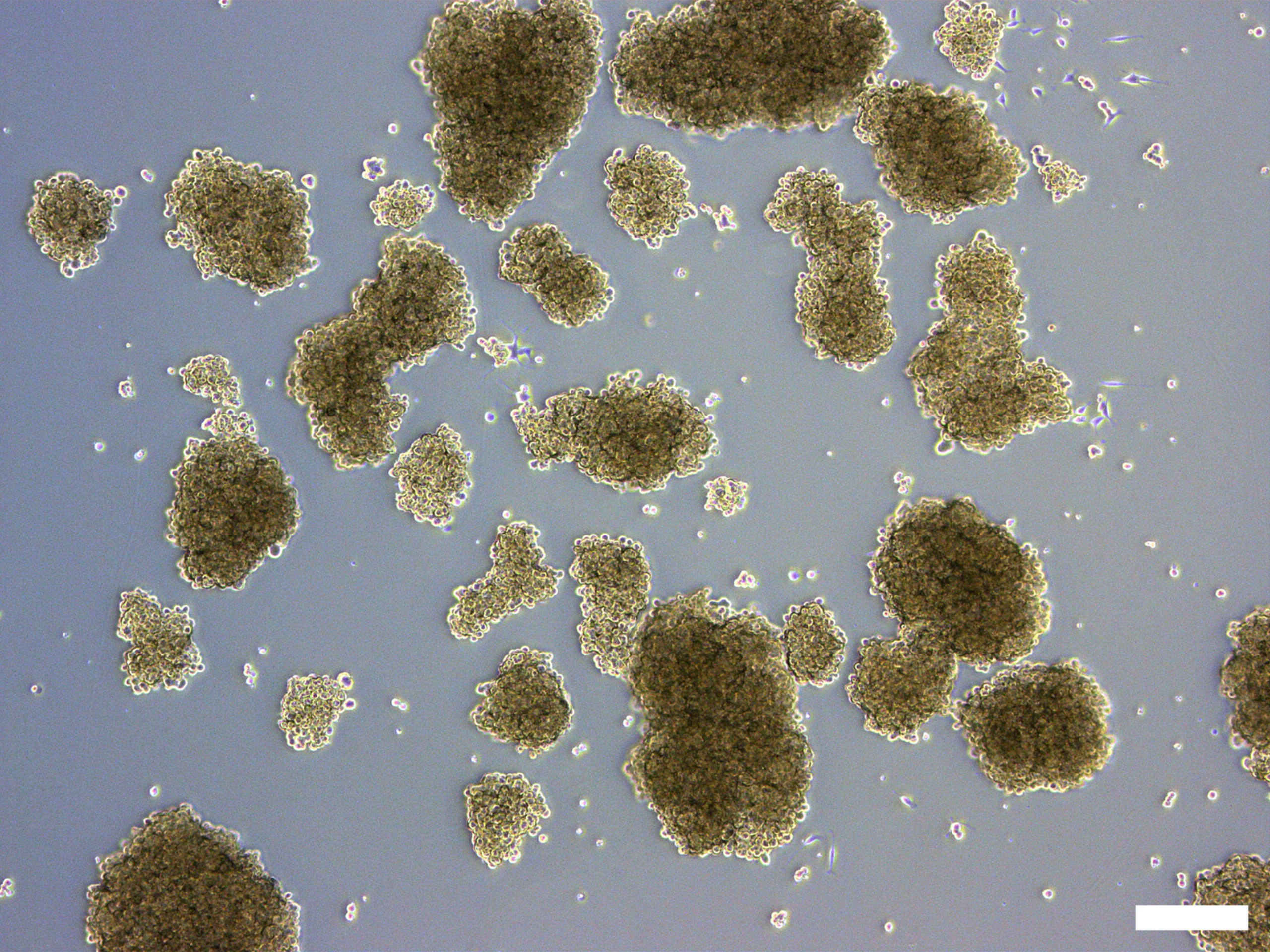

Supplement: Supplementary file 3 — Source data Fig. 1 [file 44319_2024_258_MOESM3_ESM.zip › Figure1/Fig1F/mGB1_Ctrl_SC.tif]

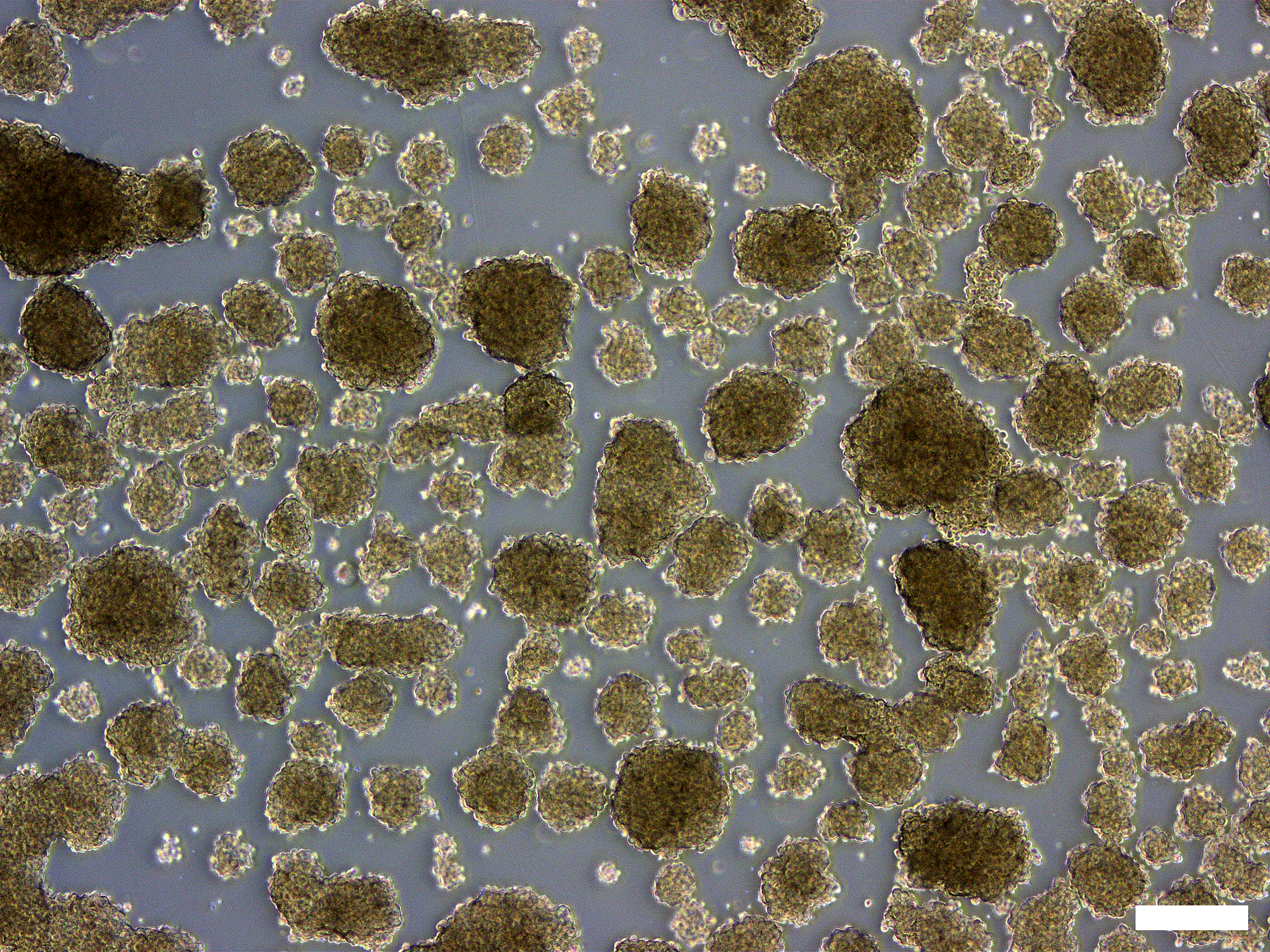

Supplement: Supplementary file 3 — Source data Fig. 1 [file 44319_2024_258_MOESM3_ESM.zip › Figure1/Fig1F/mGB1_KD1_SC.tif]

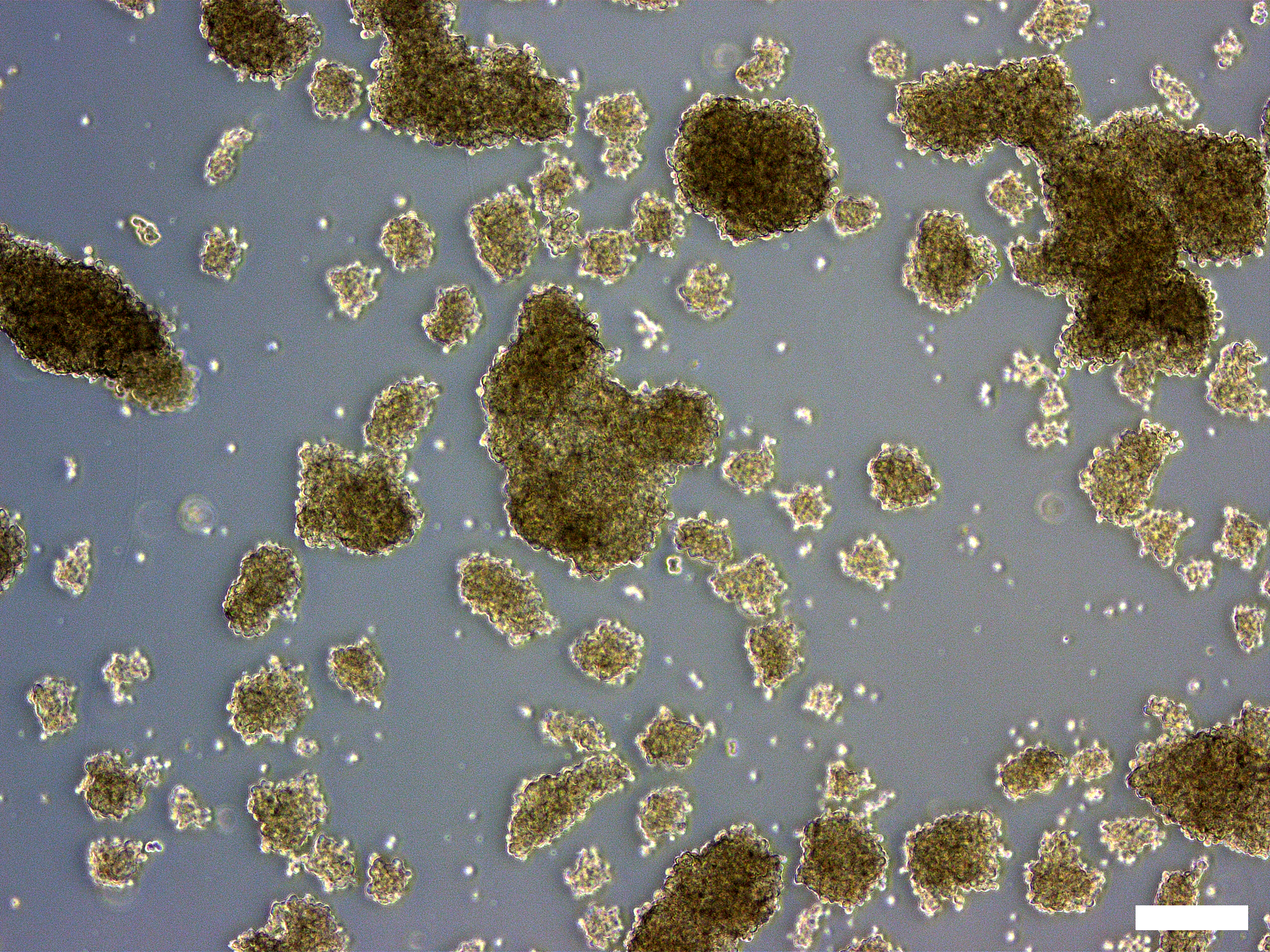

Supplement: Supplementary file 3 — Source data Fig. 1 [file 44319_2024_258_MOESM3_ESM.zip › Figure1/Fig1F/mGB1_KD2_SC.tif]

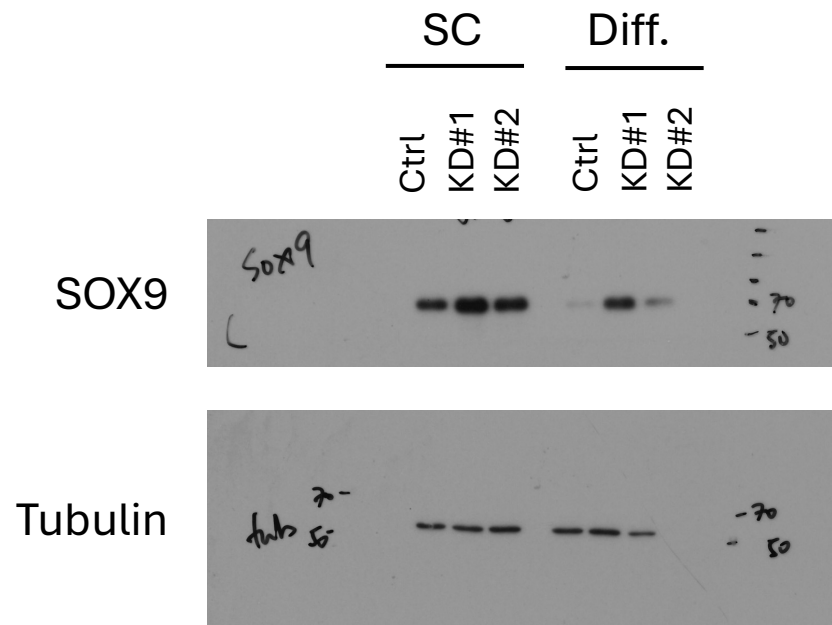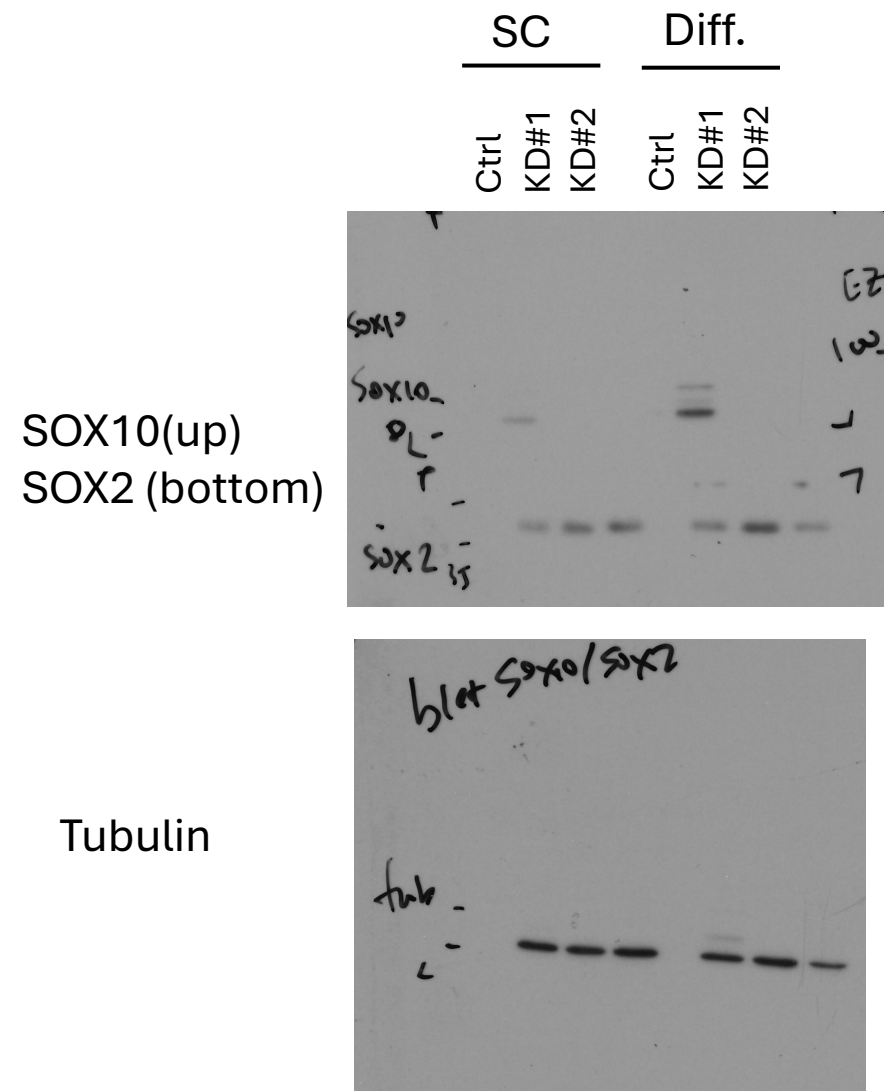

Supplement: Supplementary file 3 — Source data Fig. 1 [file 44319_2024_258_MOESM3_ESM.zip › Figure1/Fig1G/Fig1G_uncropped.pdf]

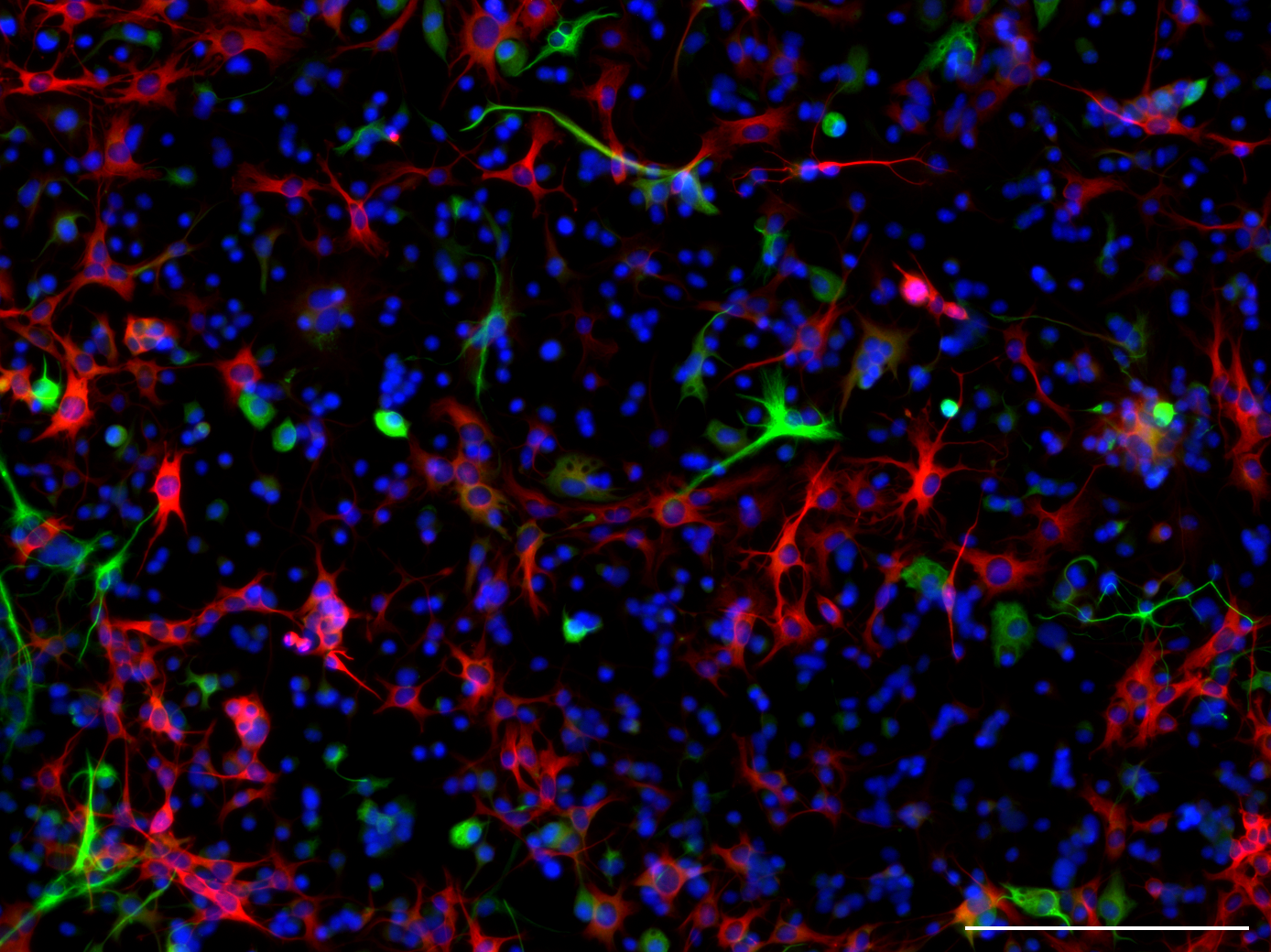

Supplement: Supplementary file 3 — Source data Fig. 1 [file 44319_2024_258_MOESM3_ESM.zip › Figure1/Fig1H/mGB1-Ctrl-Diff.png]

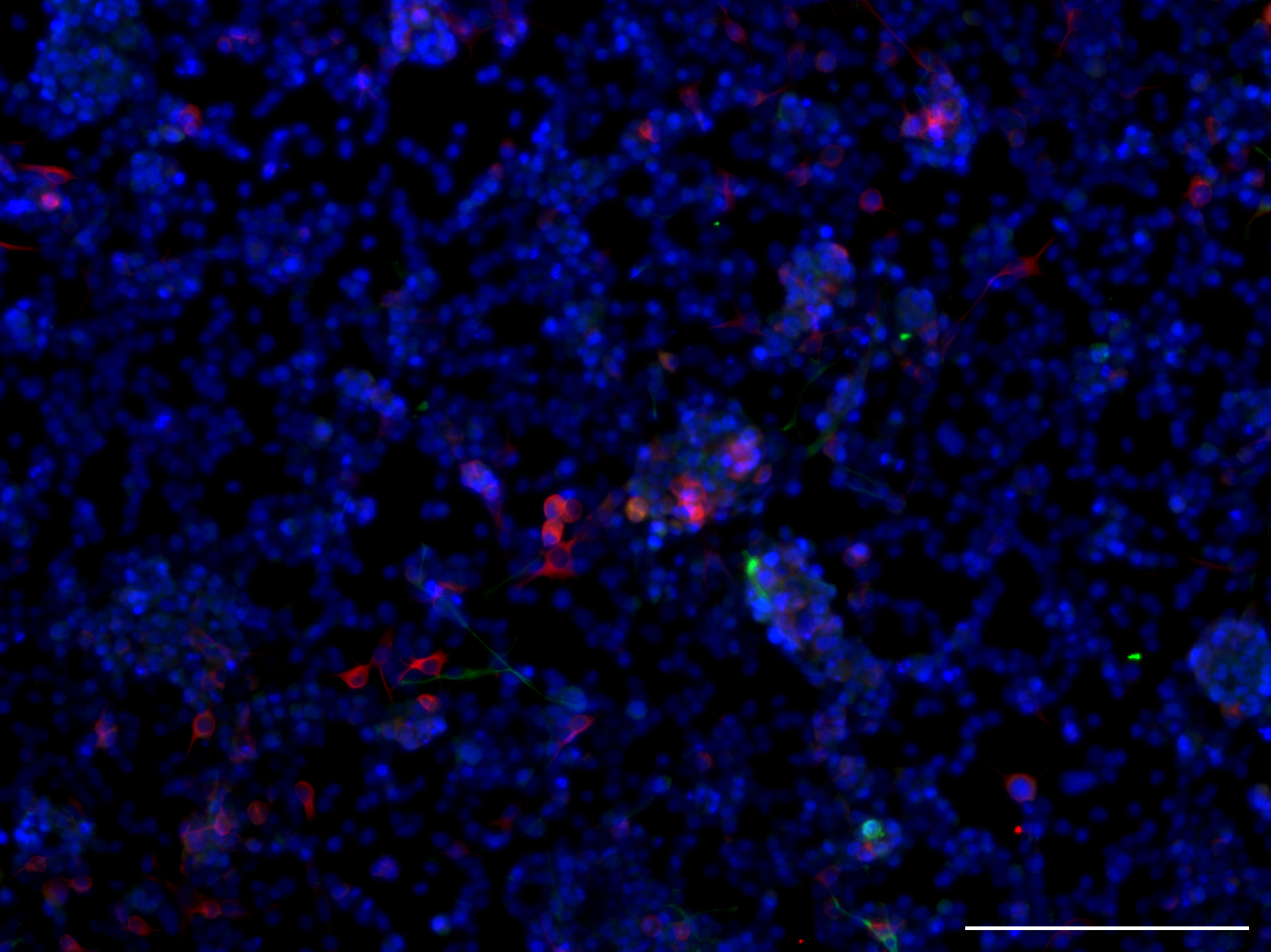

Supplement: Supplementary file 3 — Source data Fig. 1 [file 44319_2024_258_MOESM3_ESM.zip › Figure1/Fig1H/mGB1-Ctrl-SC.png]

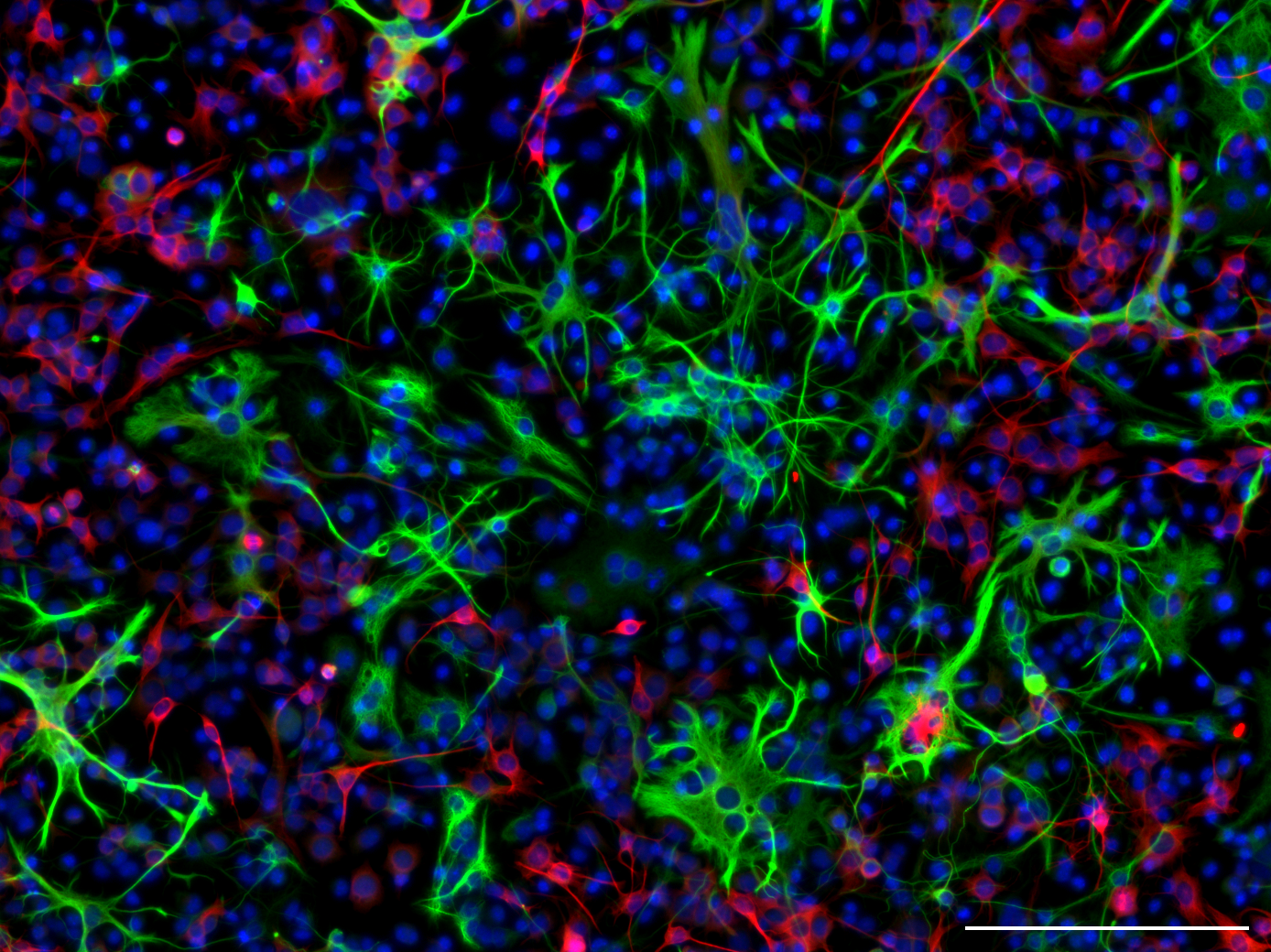

Supplement: Supplementary file 3 — Source data Fig. 1 [file 44319_2024_258_MOESM3_ESM.zip › Figure1/Fig1H/mGB1-KD1-Diff.png]

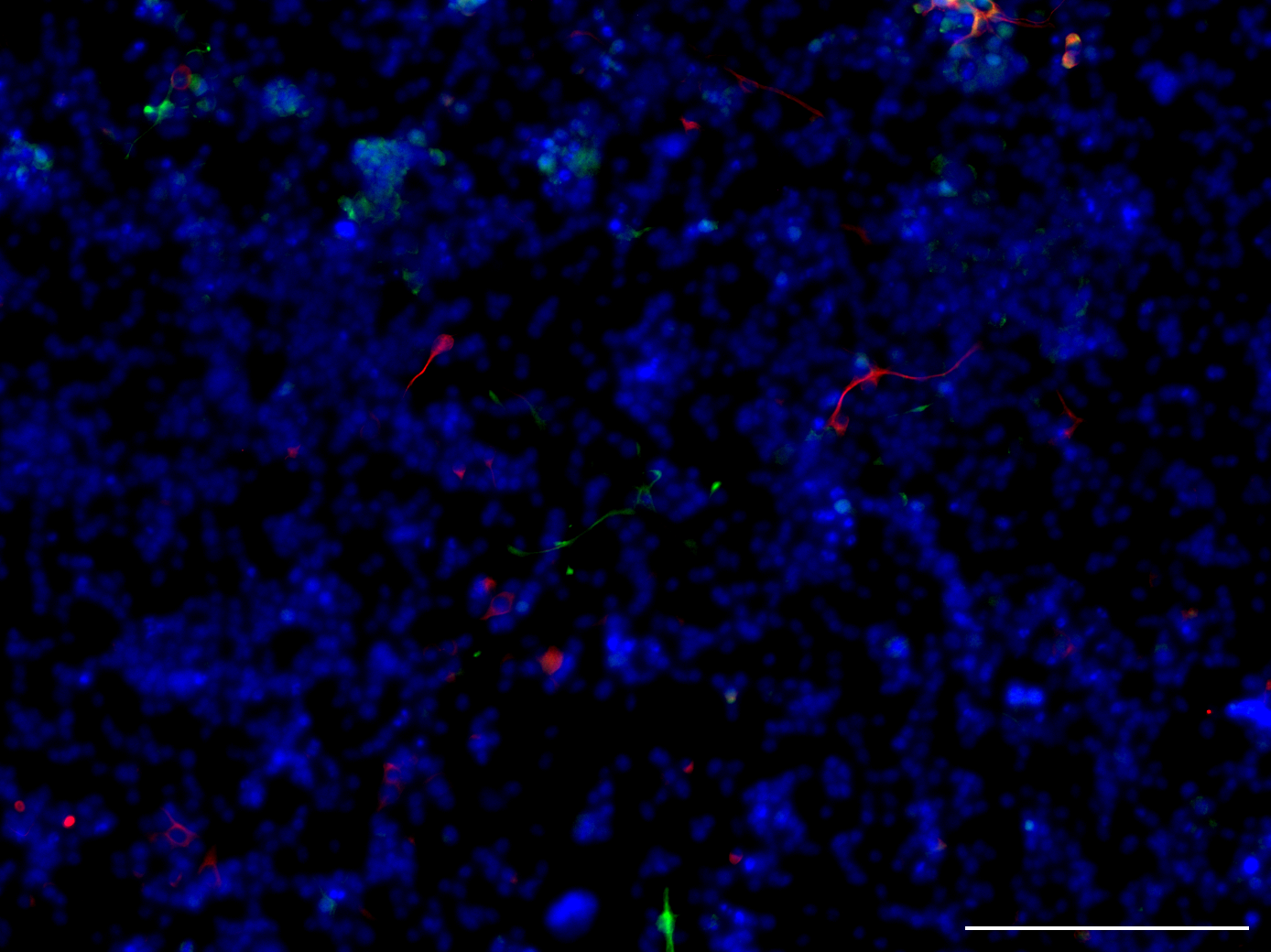

Supplement: Supplementary file 3 — Source data Fig. 1 [file 44319_2024_258_MOESM3_ESM.zip › Figure1/Fig1H/mGB1-KD1-SC.png]

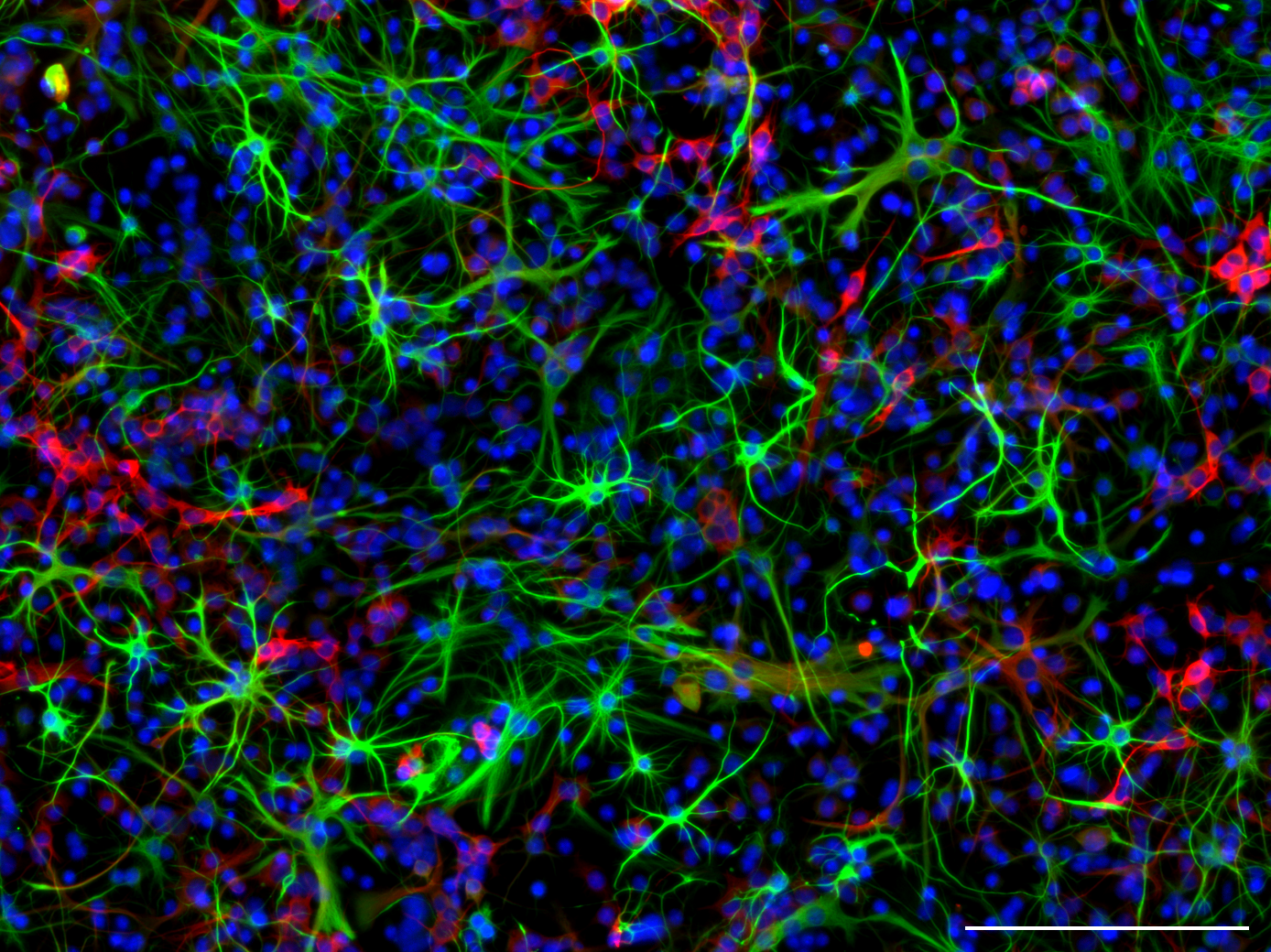

Supplement: Supplementary file 3 — Source data Fig. 1 [file 44319_2024_258_MOESM3_ESM.zip › Figure1/Fig1H/mGB1-KD2-Diff.png]

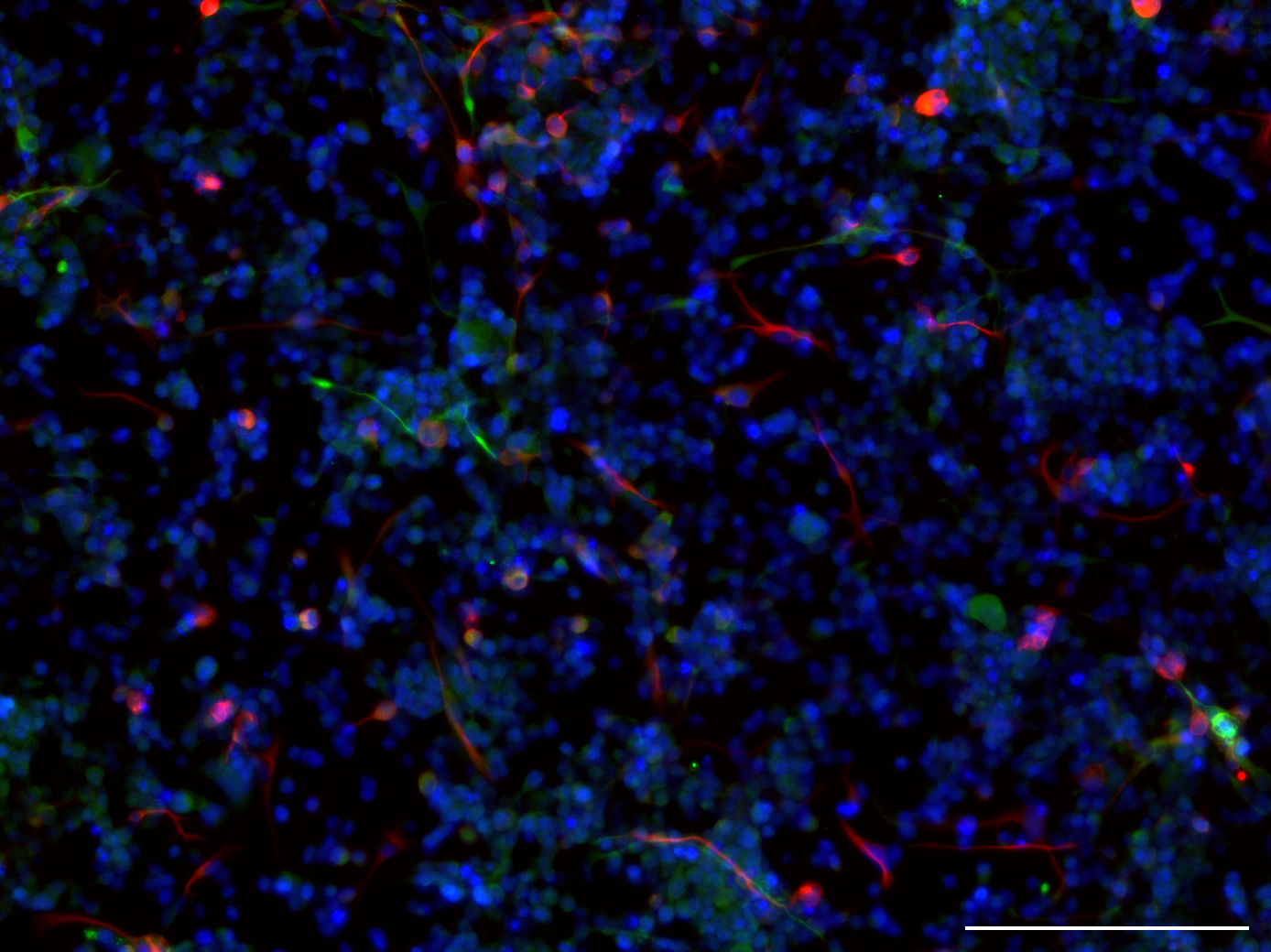

Supplement: Supplementary file 3 — Source data Fig. 1 [file 44319_2024_258_MOESM3_ESM.zip › Figure1/Fig1H/mGB1-KD2-SC.png]

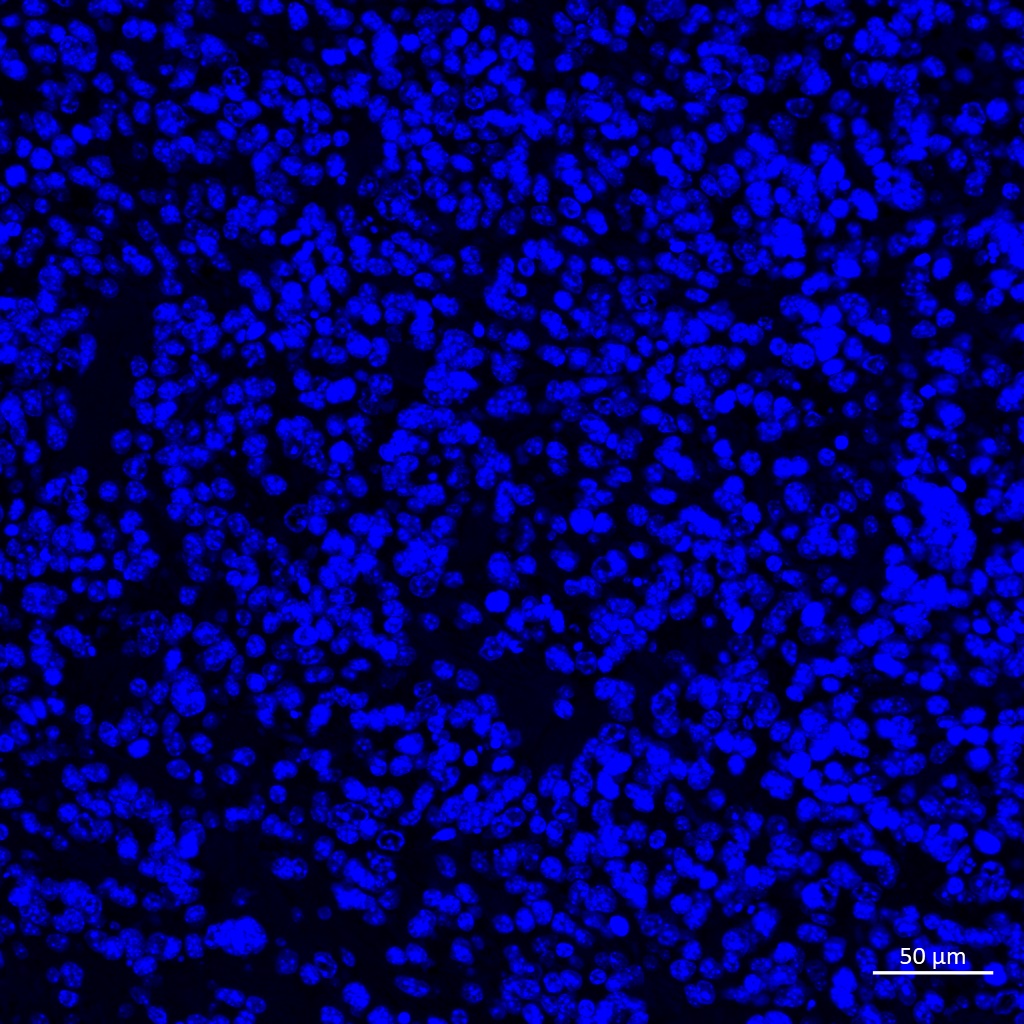

Supplement: Supplementary file 4 — Source data Fig. 2 [file 44319_2024_258_MOESM4_ESM.zip › Figure2/Fig2G/mGB1_Ctrl_tumor_DAPI.jpg]

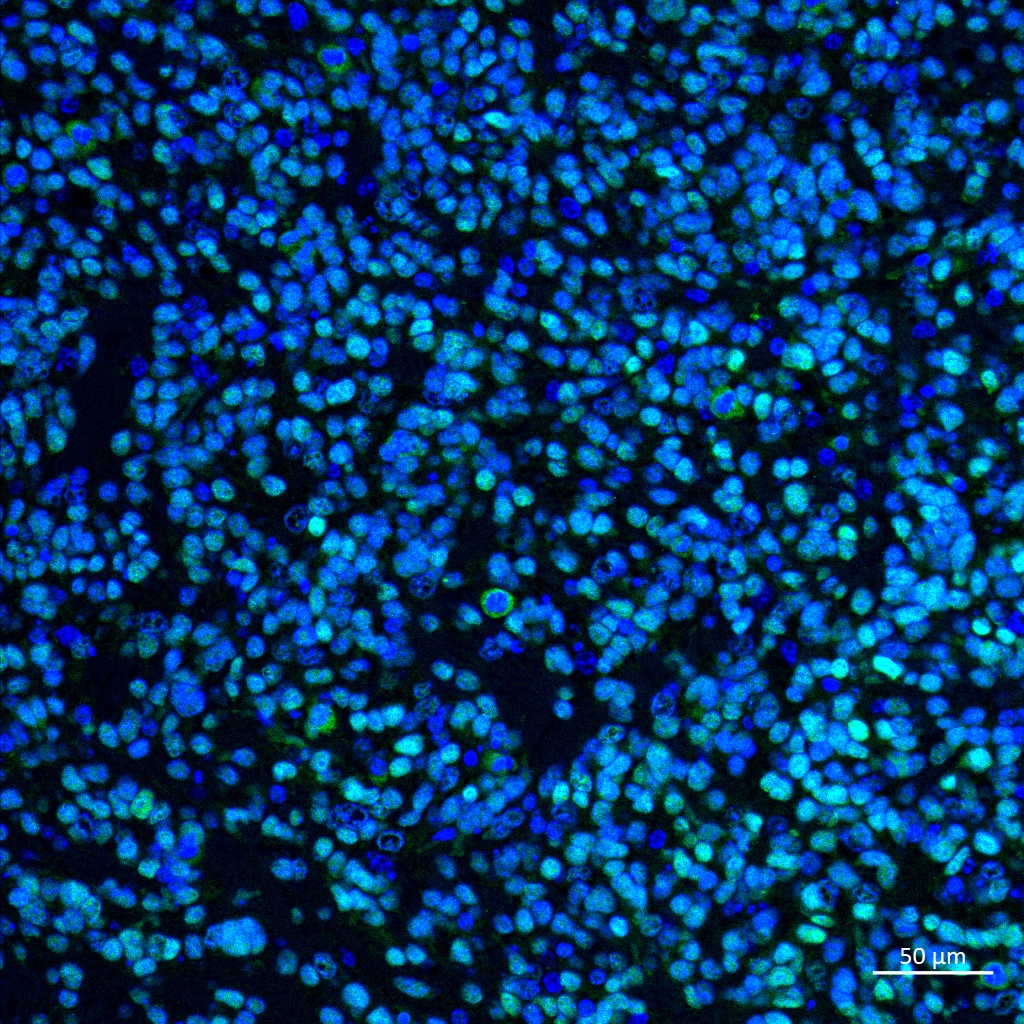

Supplement: Supplementary file 4 — Source data Fig. 2 [file 44319_2024_258_MOESM4_ESM.zip › Figure2/Fig2G/mGB1_Ctrl_tumor_Merge.jpg]

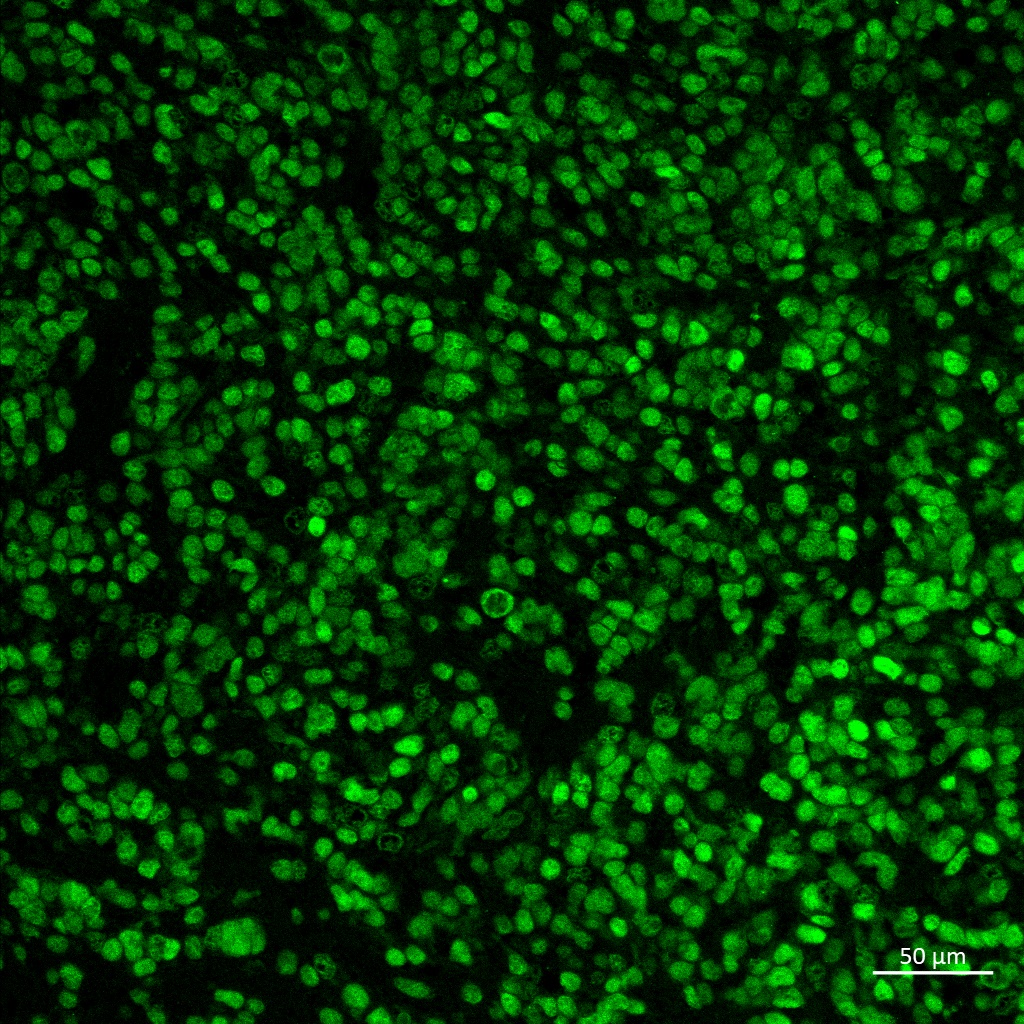

Supplement: Supplementary file 4 — Source data Fig. 2 [file 44319_2024_258_MOESM4_ESM.zip › Figure2/Fig2G/mGB1_Ctrl_tumor_Sox2.jpg]

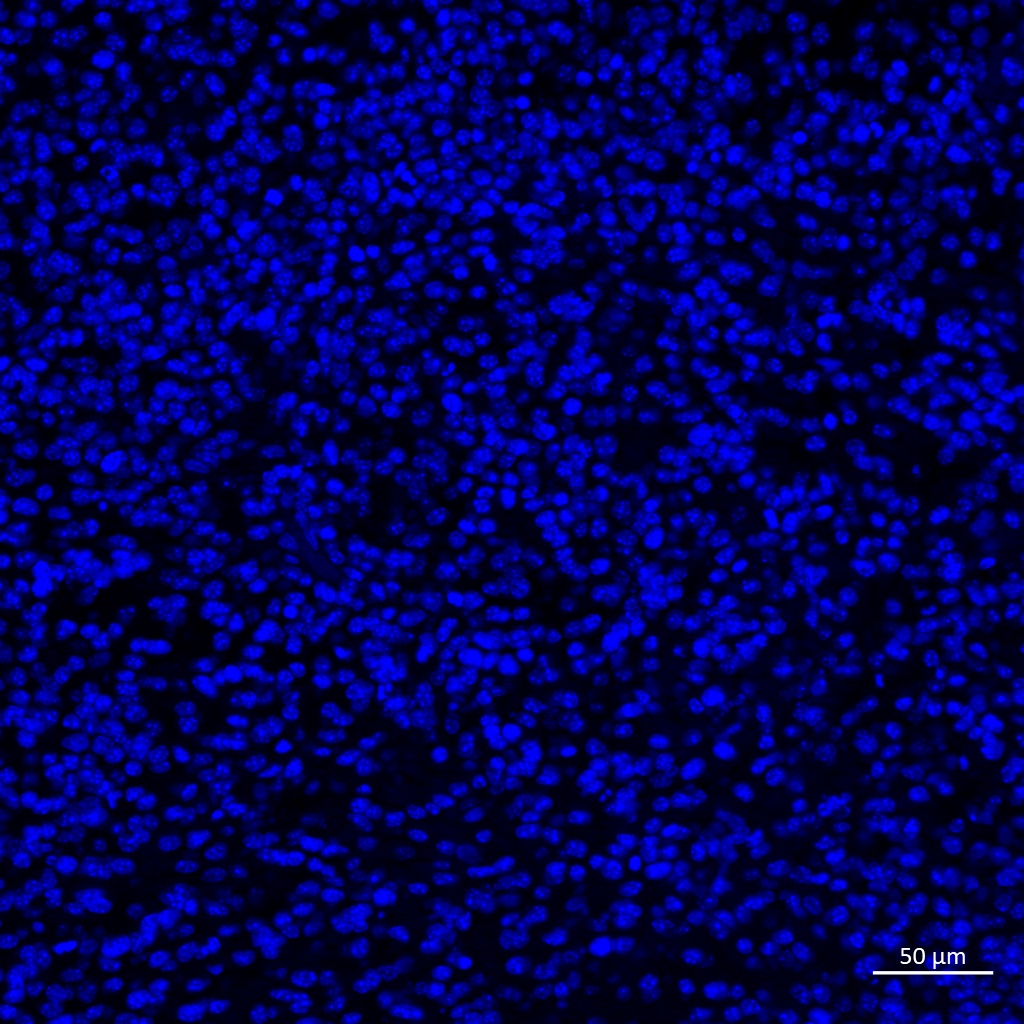

Supplement: Supplementary file 4 — Source data Fig. 2 [file 44319_2024_258_MOESM4_ESM.zip › Figure2/Fig2G/mGB1_KD_tumor_DAPI.jpg]

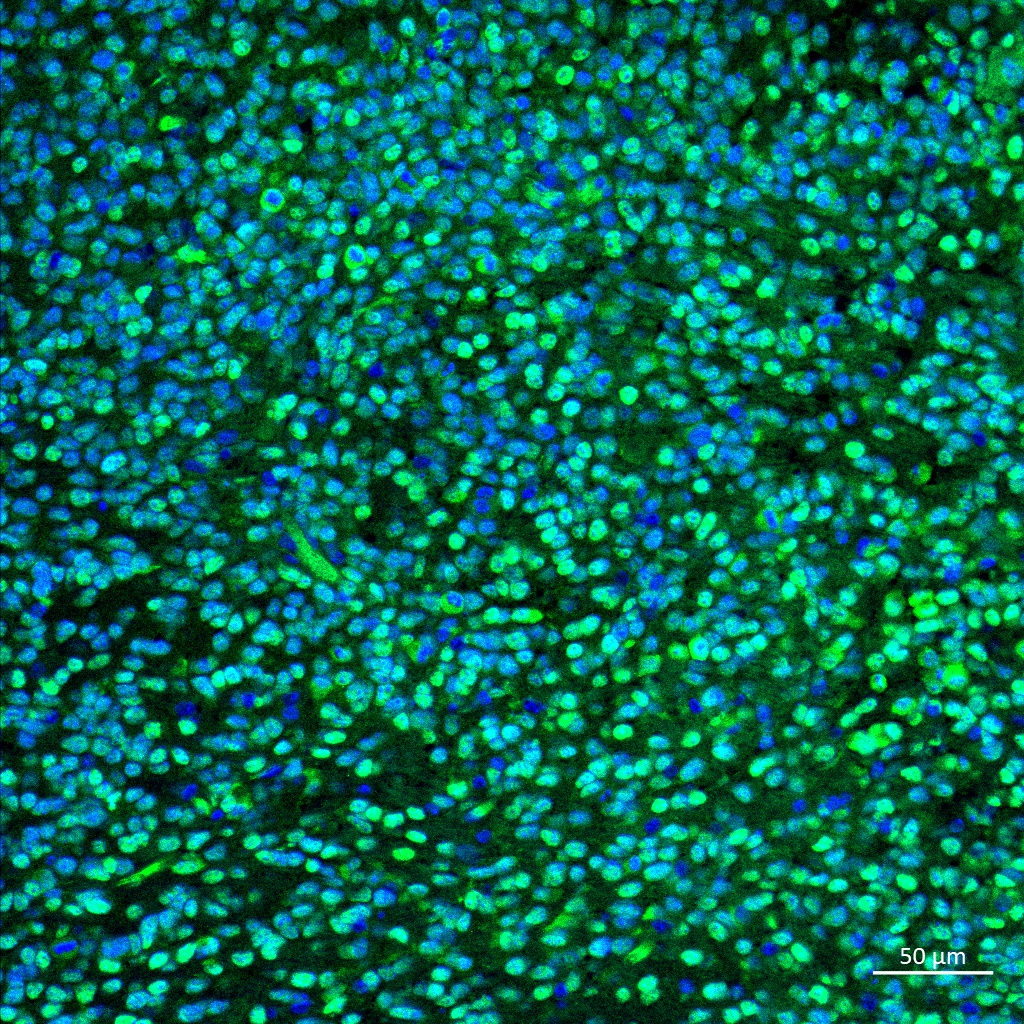

Supplement: Supplementary file 4 — Source data Fig. 2 [file 44319_2024_258_MOESM4_ESM.zip › Figure2/Fig2G/mGB1_KD_tumor_Merge.jpg]

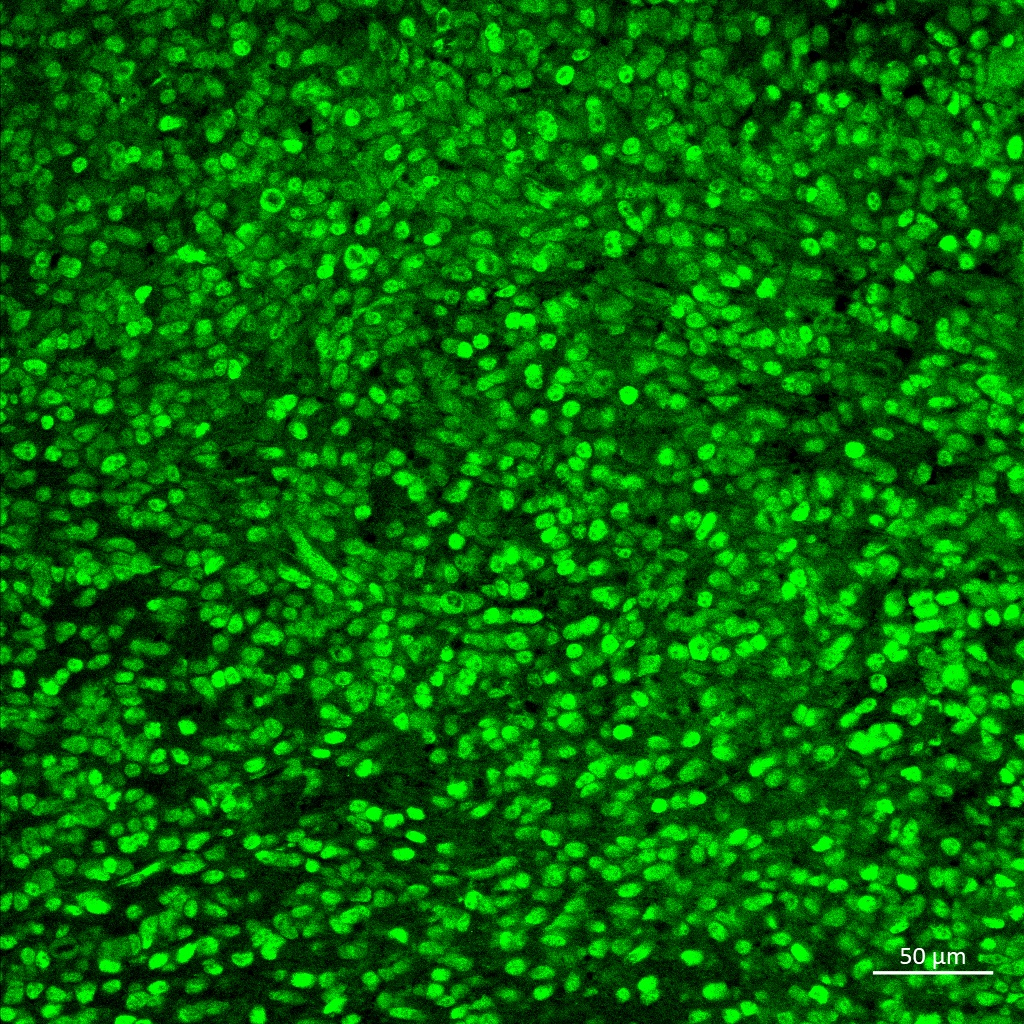

Supplement: Supplementary file 4 — Source data Fig. 2 [file 44319_2024_258_MOESM4_ESM.zip › Figure2/Fig2G/mGB1_KD_tumor_Sox2.jpg]

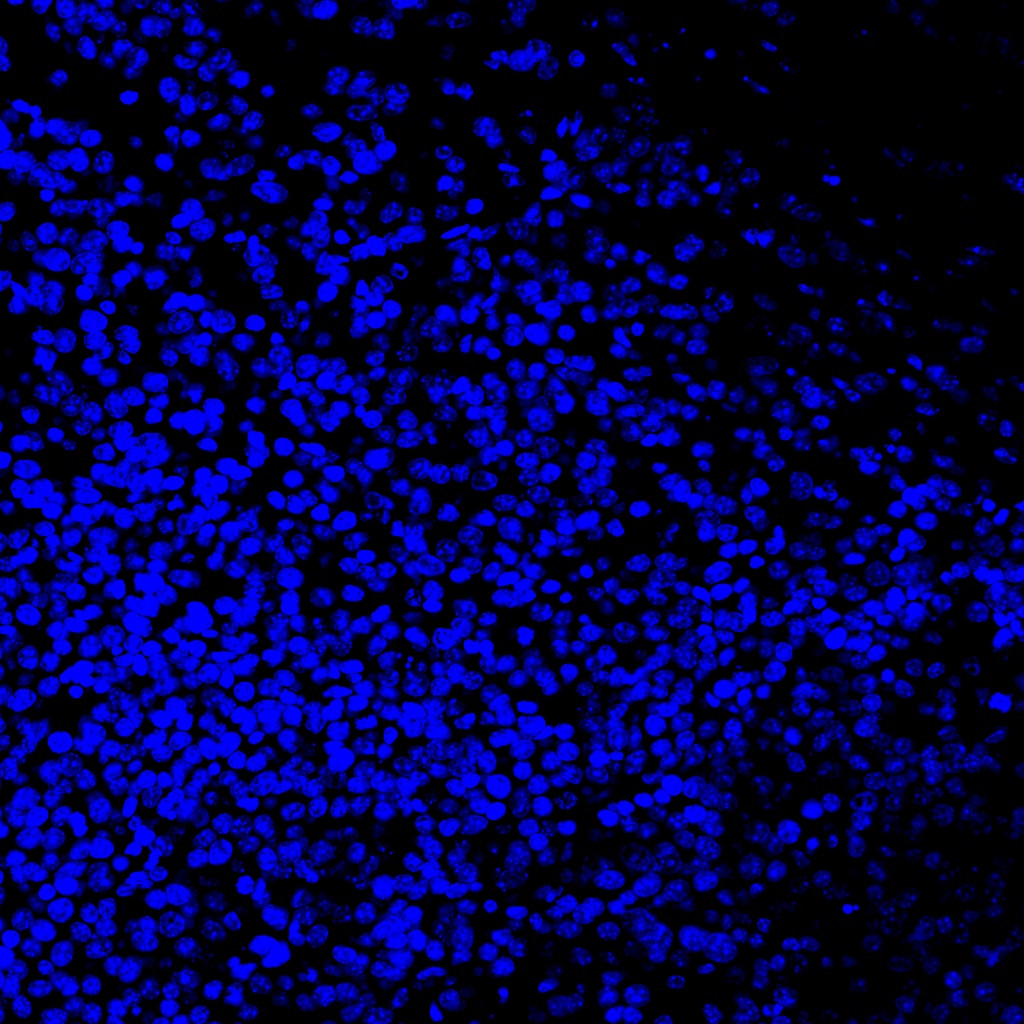

Supplement: Supplementary file 4 — Source data Fig. 2 [file 44319_2024_258_MOESM4_ESM.zip › Figure2/Fig2H/mGB1_Ctrl_tumor_DAPI.jpg]

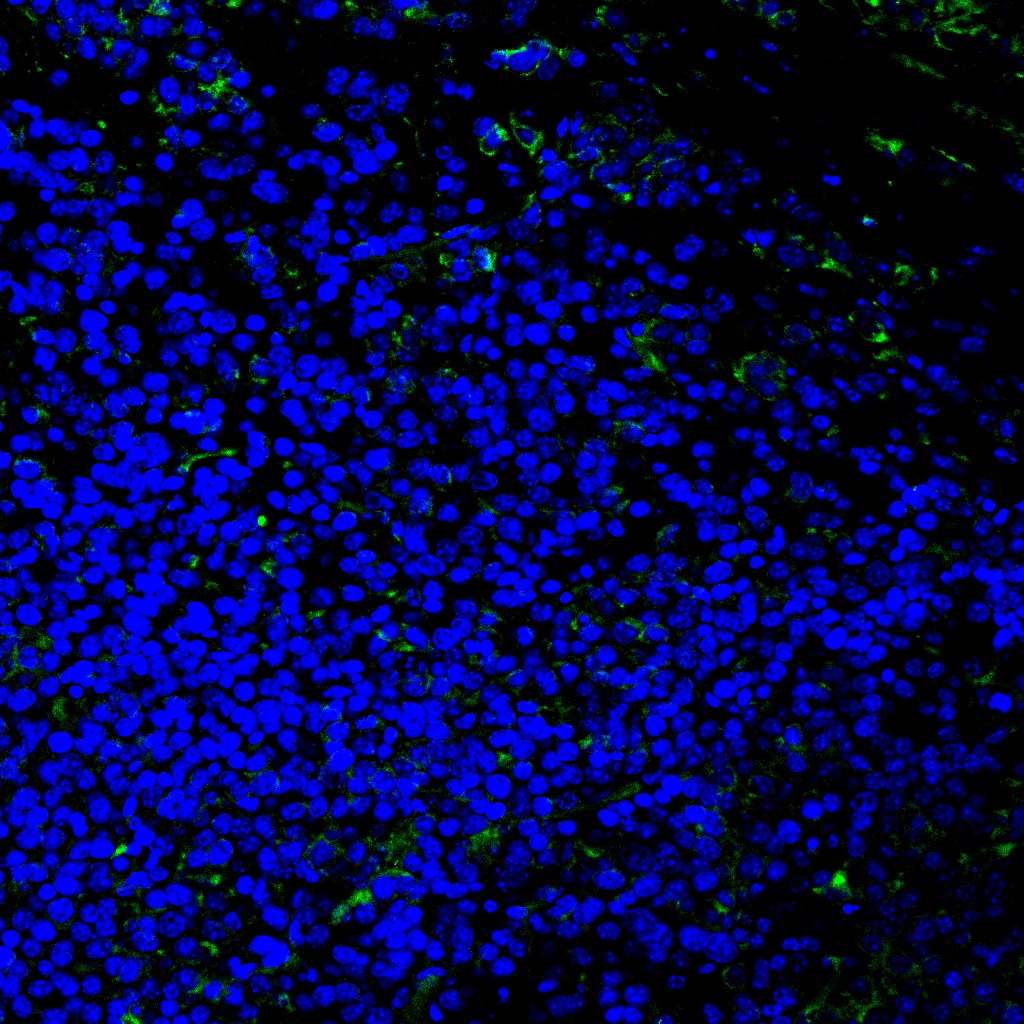

Supplement: Supplementary file 4 — Source data Fig. 2 [file 44319_2024_258_MOESM4_ESM.zip › Figure2/Fig2H/mGB1_Ctrl_tumor_Merge.jpg]

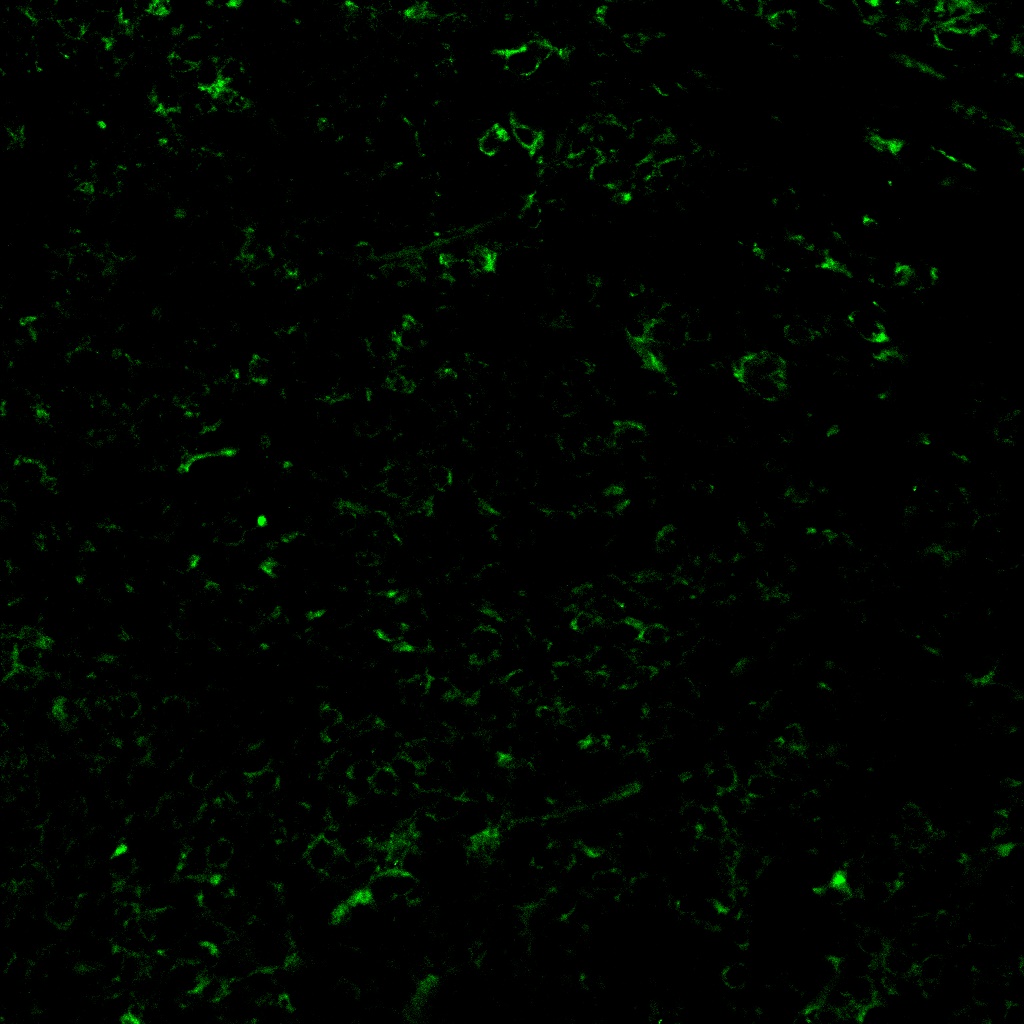

Supplement: Supplementary file 4 — Source data Fig. 2 [file 44319_2024_258_MOESM4_ESM.zip › Figure2/Fig2H/mGB1_Ctrl_tumor_Nestin.jpg]

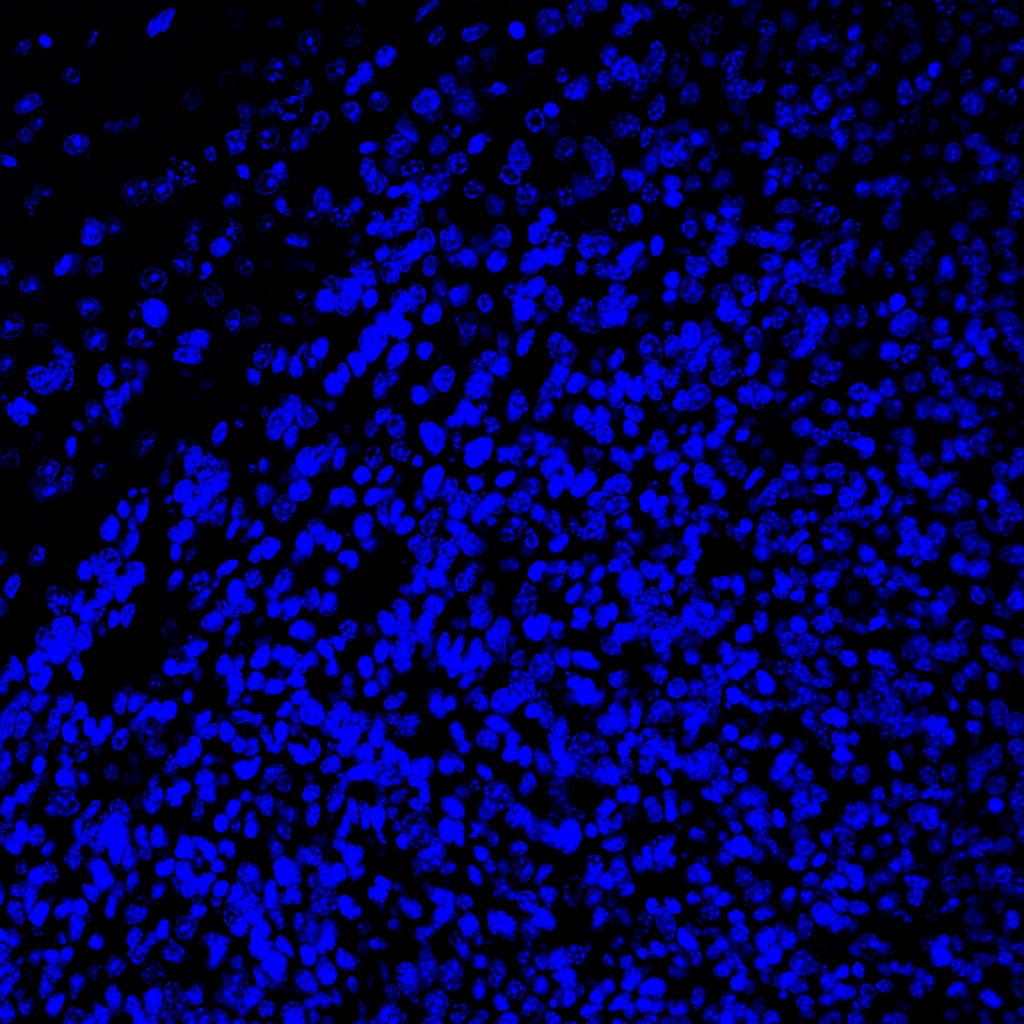

Supplement: Supplementary file 4 — Source data Fig. 2 [file 44319_2024_258_MOESM4_ESM.zip › Figure2/Fig2H/mGB1_KD_tumor_DAPI.jpg]

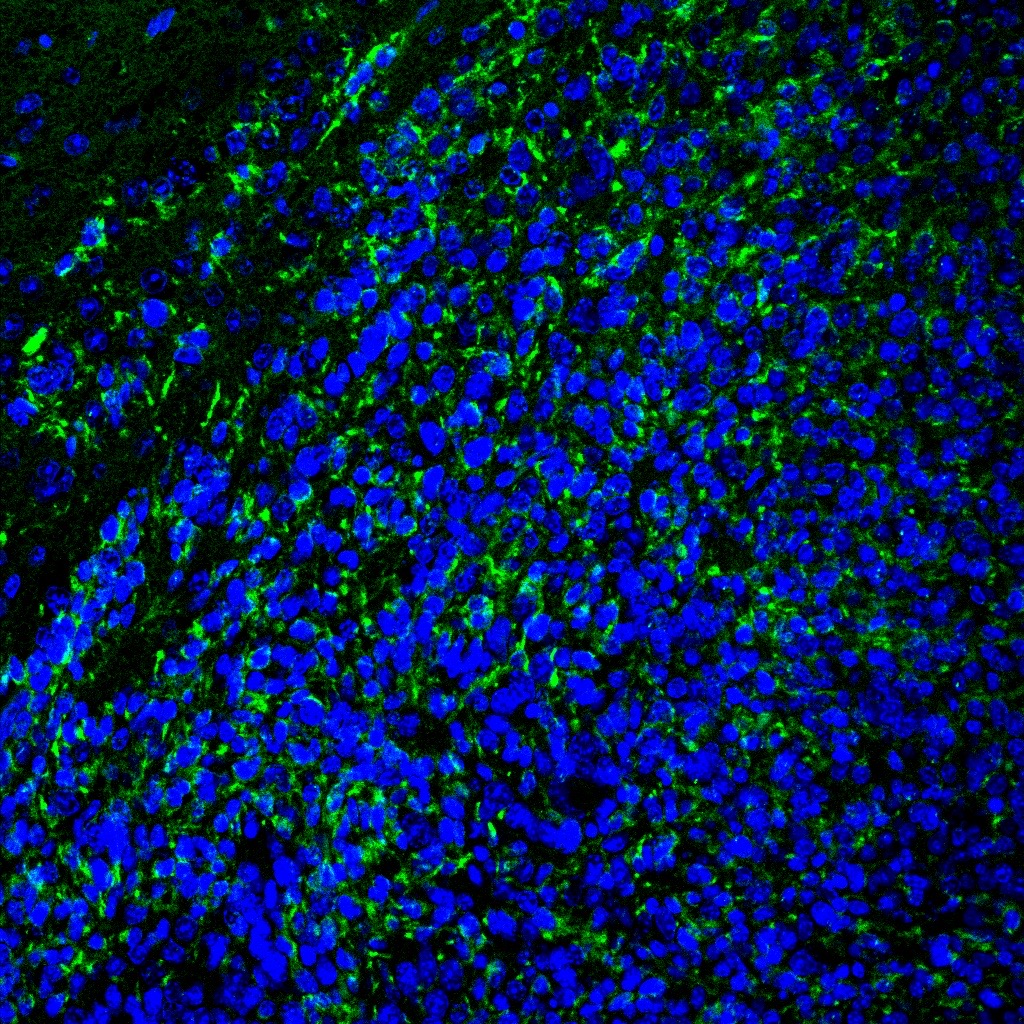

Supplement: Supplementary file 4 — Source data Fig. 2 [file 44319_2024_258_MOESM4_ESM.zip › Figure2/Fig2H/mGB1_KD_tumor_Merge.jpg]

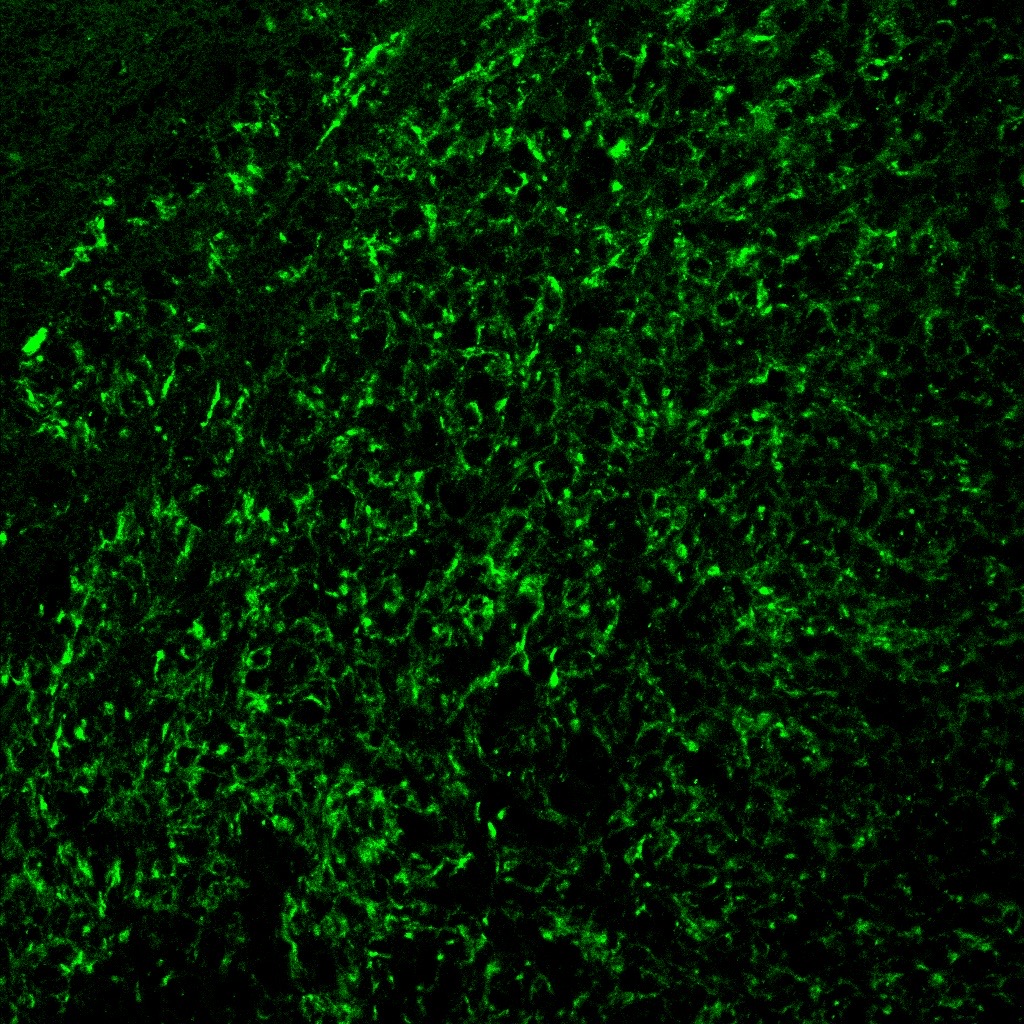

Supplement: Supplementary file 4 — Source data Fig. 2 [file 44319_2024_258_MOESM4_ESM.zip › Figure2/Fig2H/mGB1_KD_tumor_Nestin.jpg]

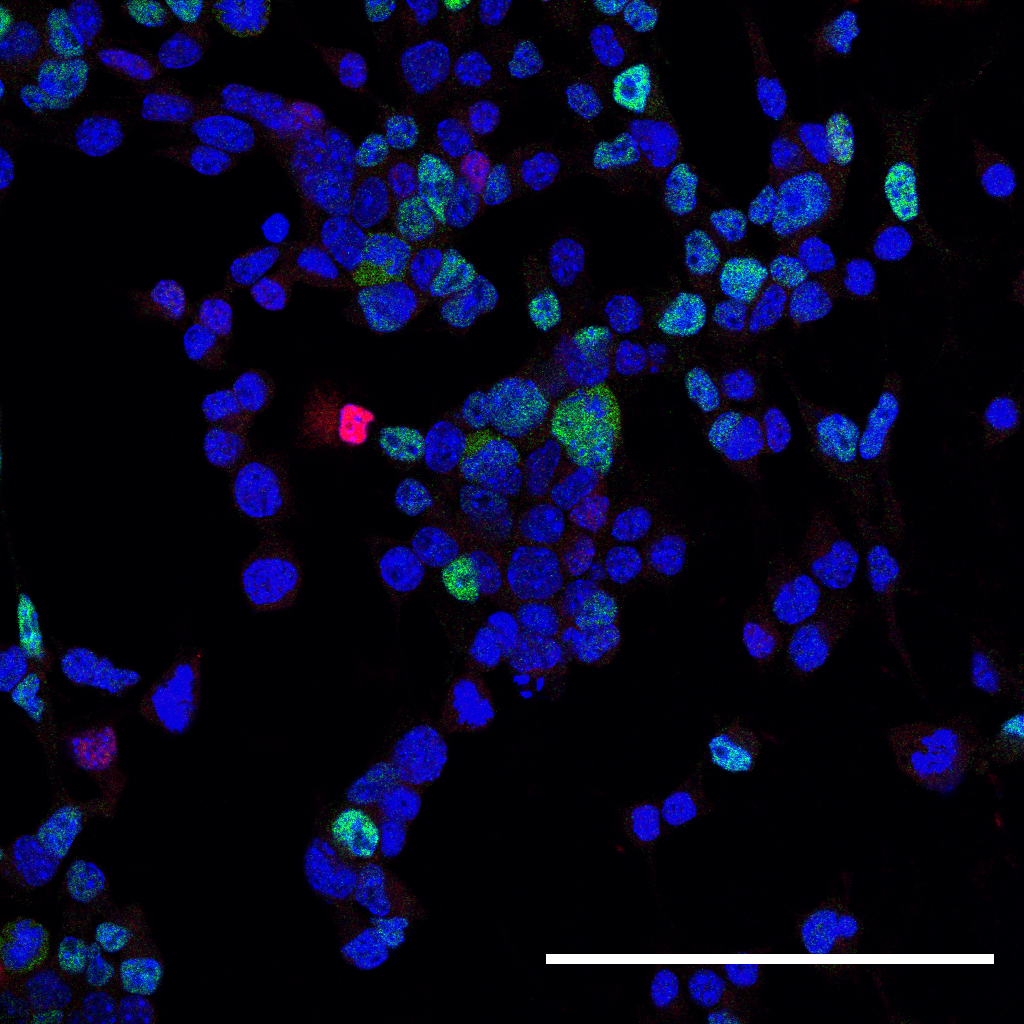

Supplement: Supplementary file 5 — Source data Fig. 3 [file 44319_2024_258_MOESM5_ESM.zip › Figure3/Fig3D/mGB1-Ctrl-merge.tif]

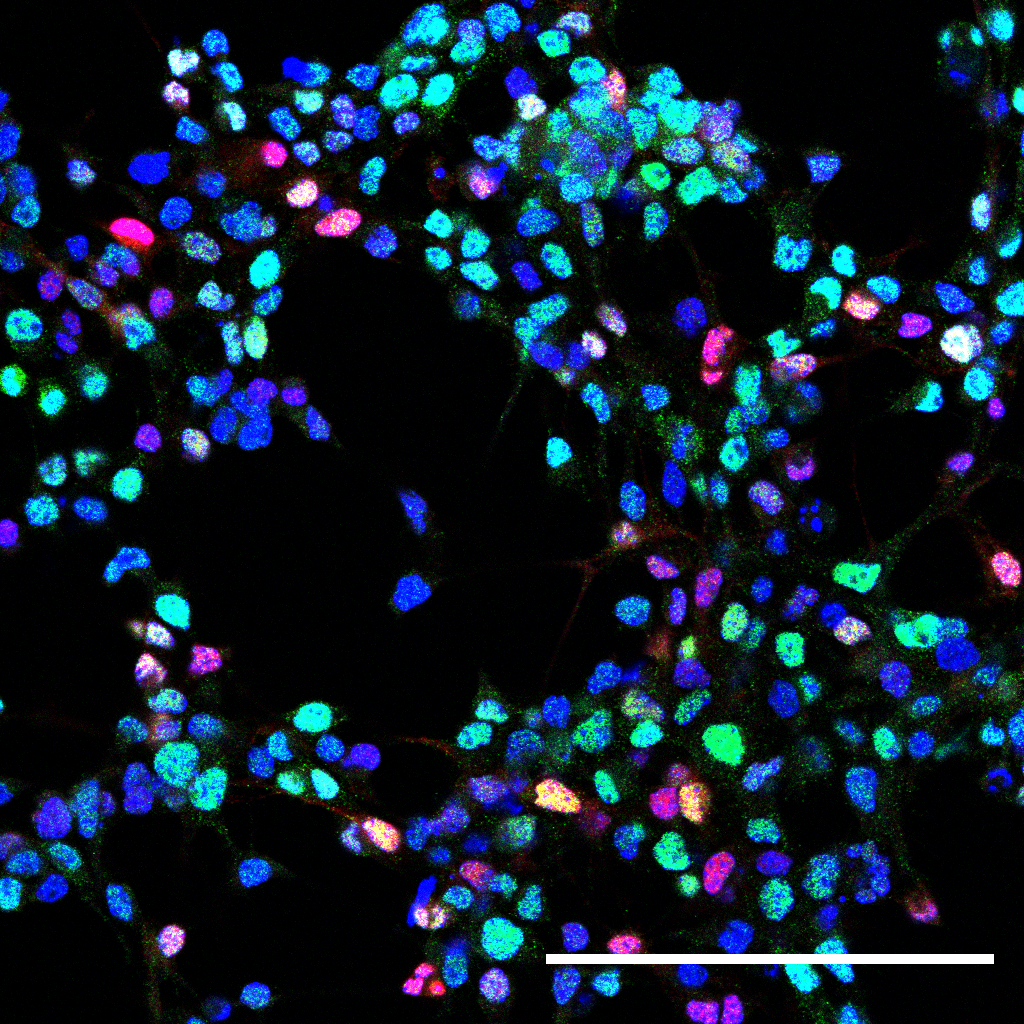

Supplement: Supplementary file 5 — Source data Fig. 3 [file 44319_2024_258_MOESM5_ESM.zip › Figure3/Fig3D/mGB1-KD-merge.tif]

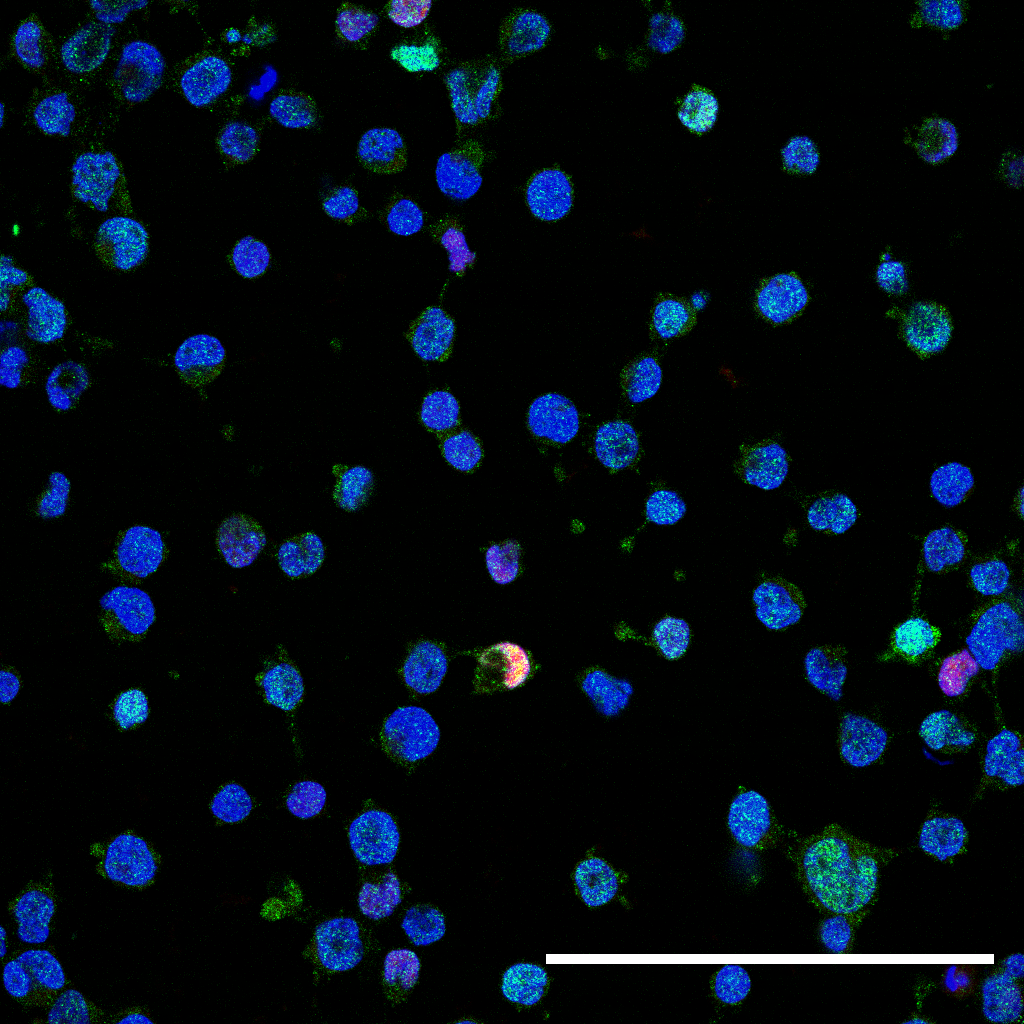

Supplement: Supplementary file 5 — Source data Fig. 3 [file 44319_2024_258_MOESM5_ESM.zip › Figure3/Fig3F/NCH421k/NCH421k-Parental-merge.tif]

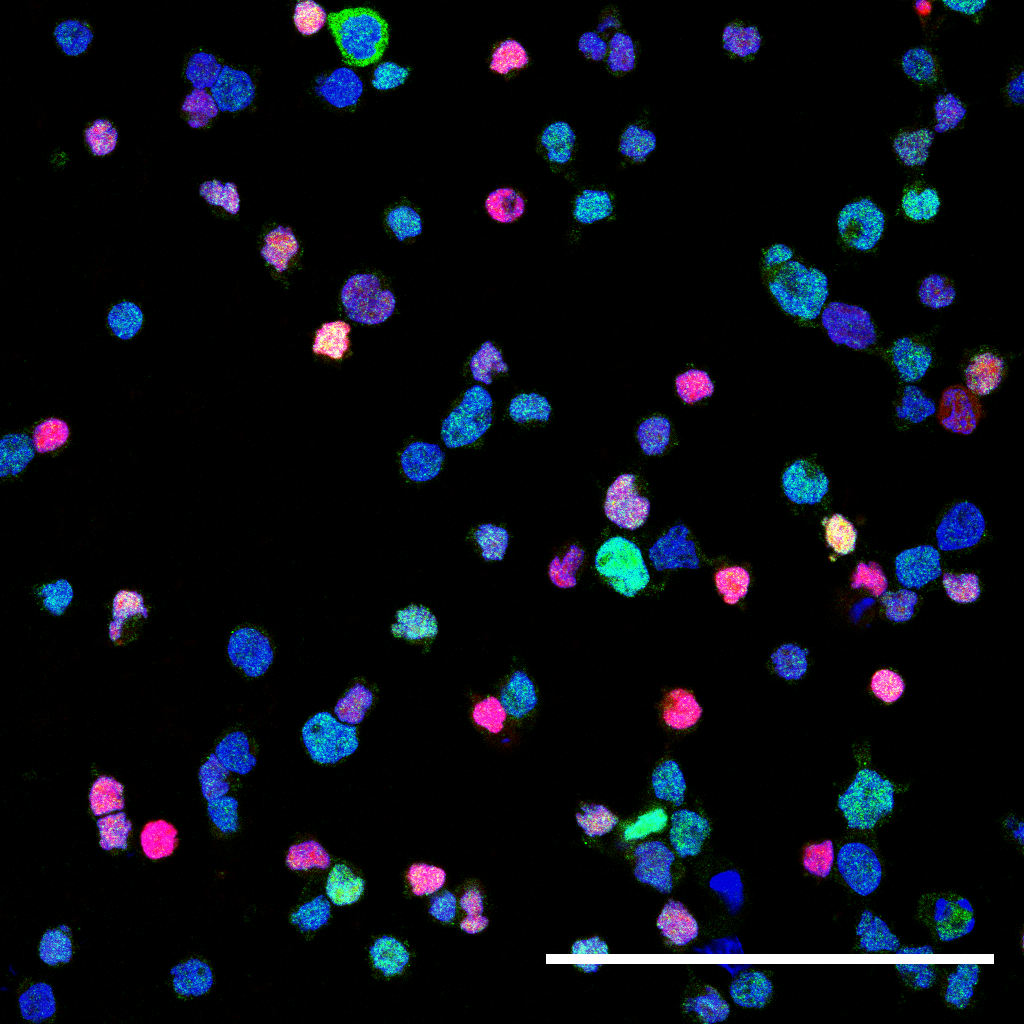

Supplement: Supplementary file 5 — Source data Fig. 3 [file 44319_2024_258_MOESM5_ESM.zip › Figure3/Fig3F/NCH421k/NCH421k-Recovered-merge.tif]

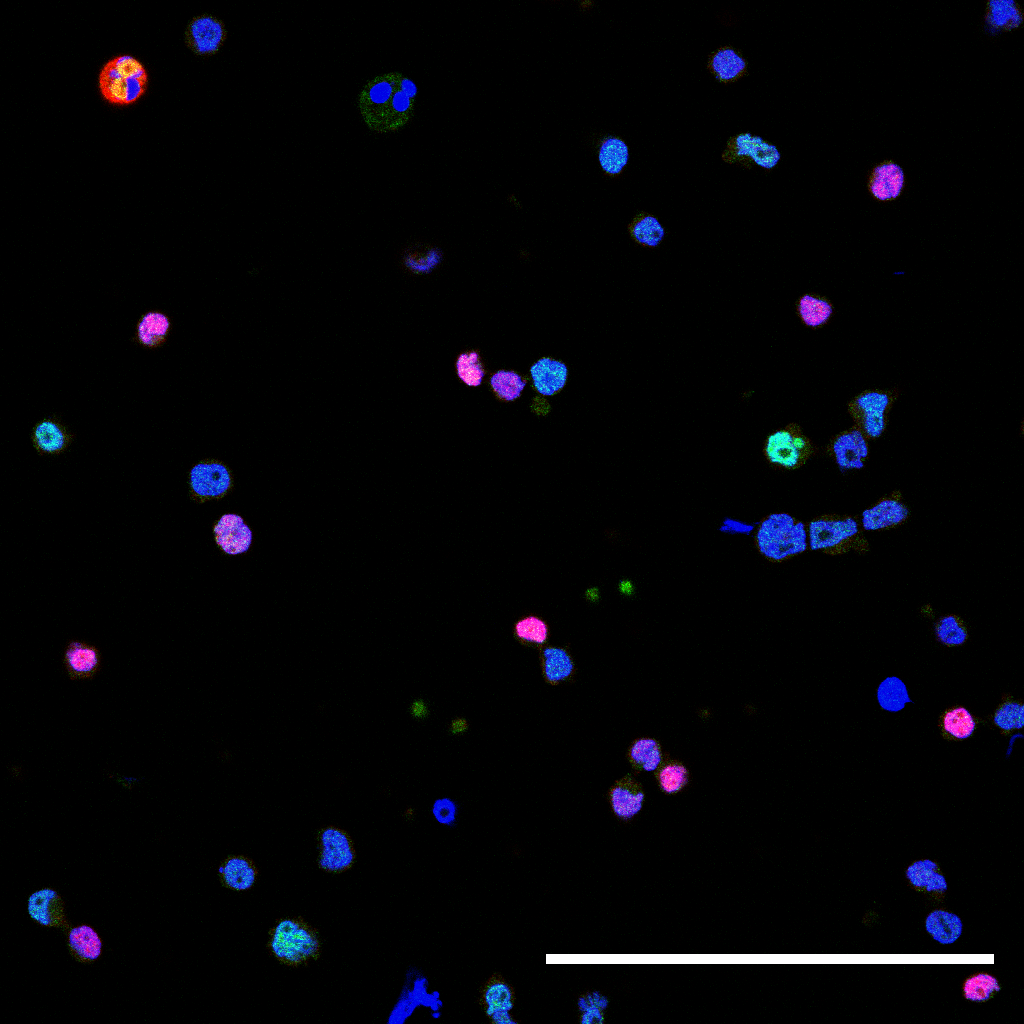

Supplement: Supplementary file 5 — Source data Fig. 3 [file 44319_2024_258_MOESM5_ESM.zip › Figure3/Fig3F/NCH441/NCH441-Parental-merge.tif]

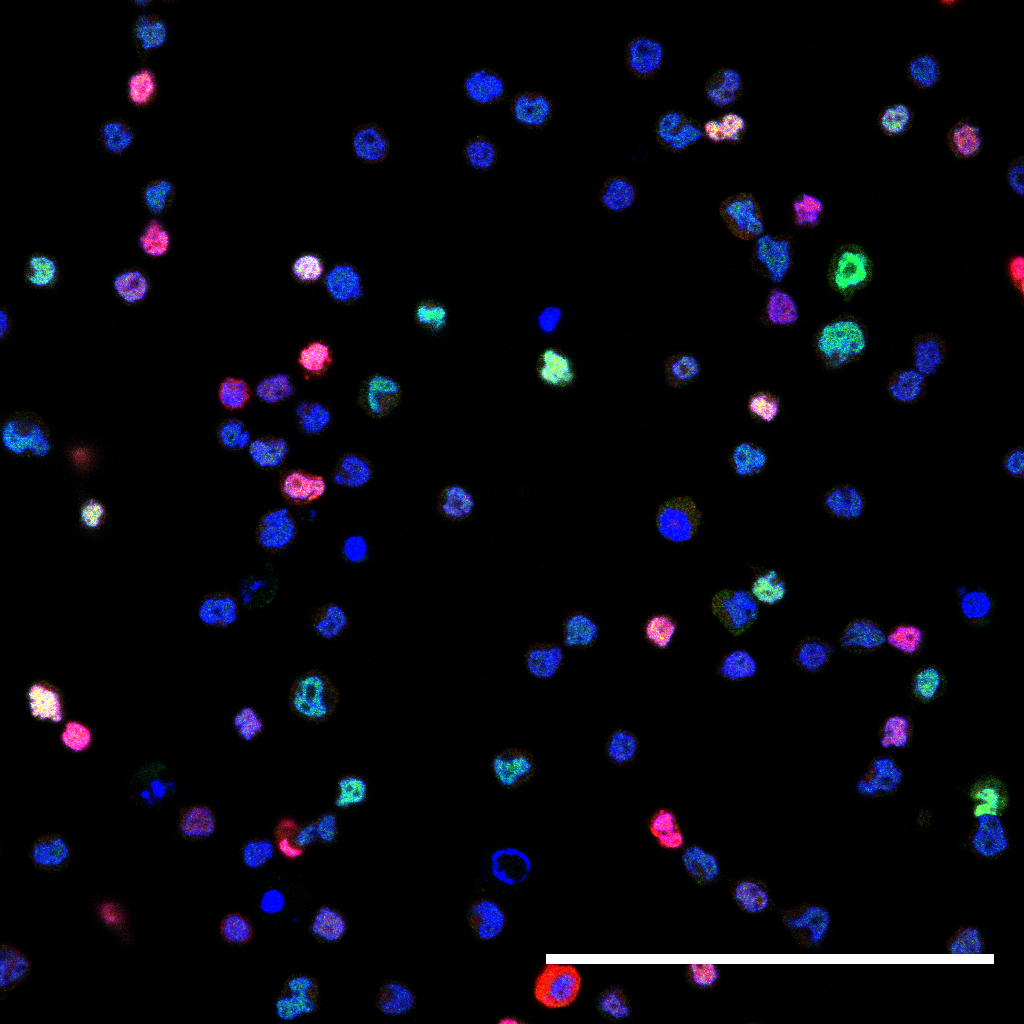

Supplement: Supplementary file 5 — Source data Fig. 3 [file 44319_2024_258_MOESM5_ESM.zip › Figure3/Fig3F/NCH441/NCH441-Recovered-merge.tif]

Fig5A

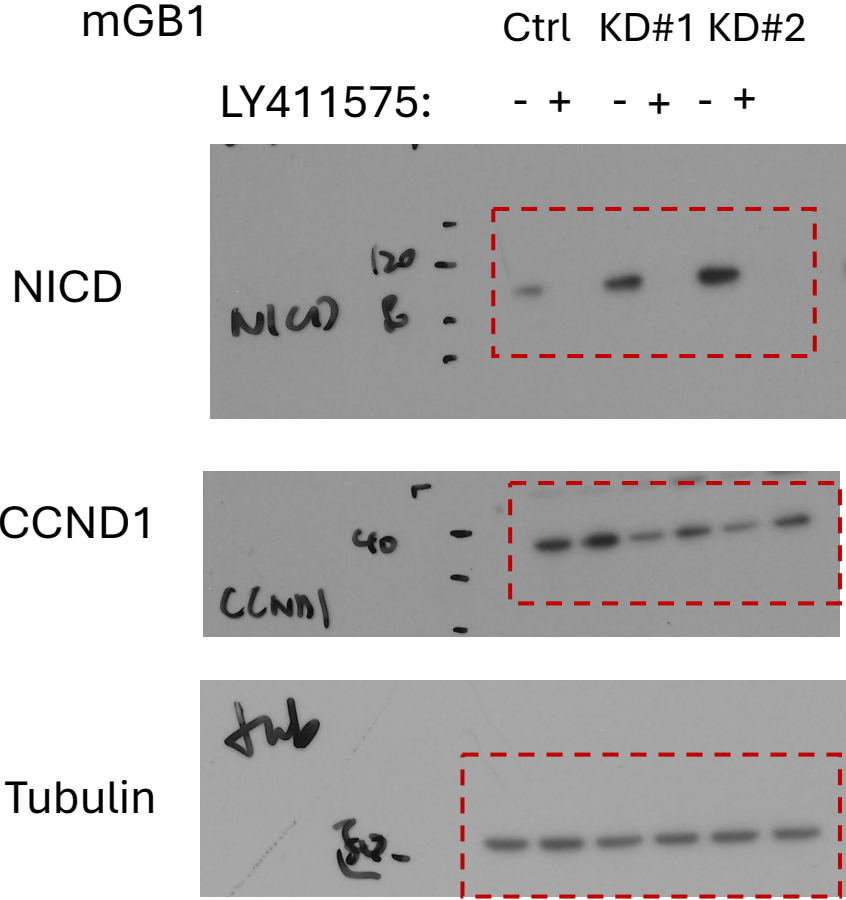

Supplement: Supplementary file 6 — Source data Fig. 4 [file 44319_2024_258_MOESM6_ESM.zip › Figure4/Fig4A/Fig4A_uncropped_blots.pdf]

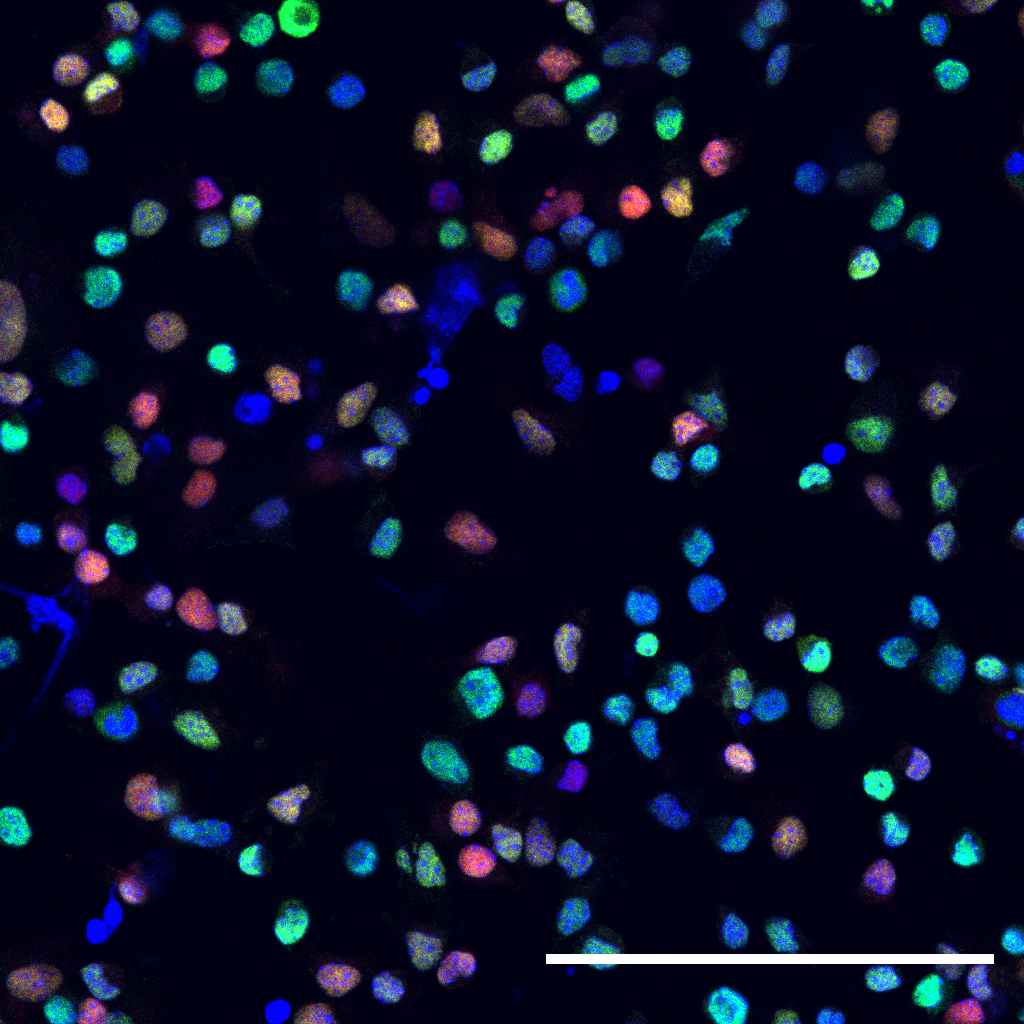

Supplement: Supplementary file 6 — Source data Fig. 4 [file 44319_2024_258_MOESM6_ESM.zip › Figure4/Fig4C/mGB1-KD-DMSO-merge.tif]

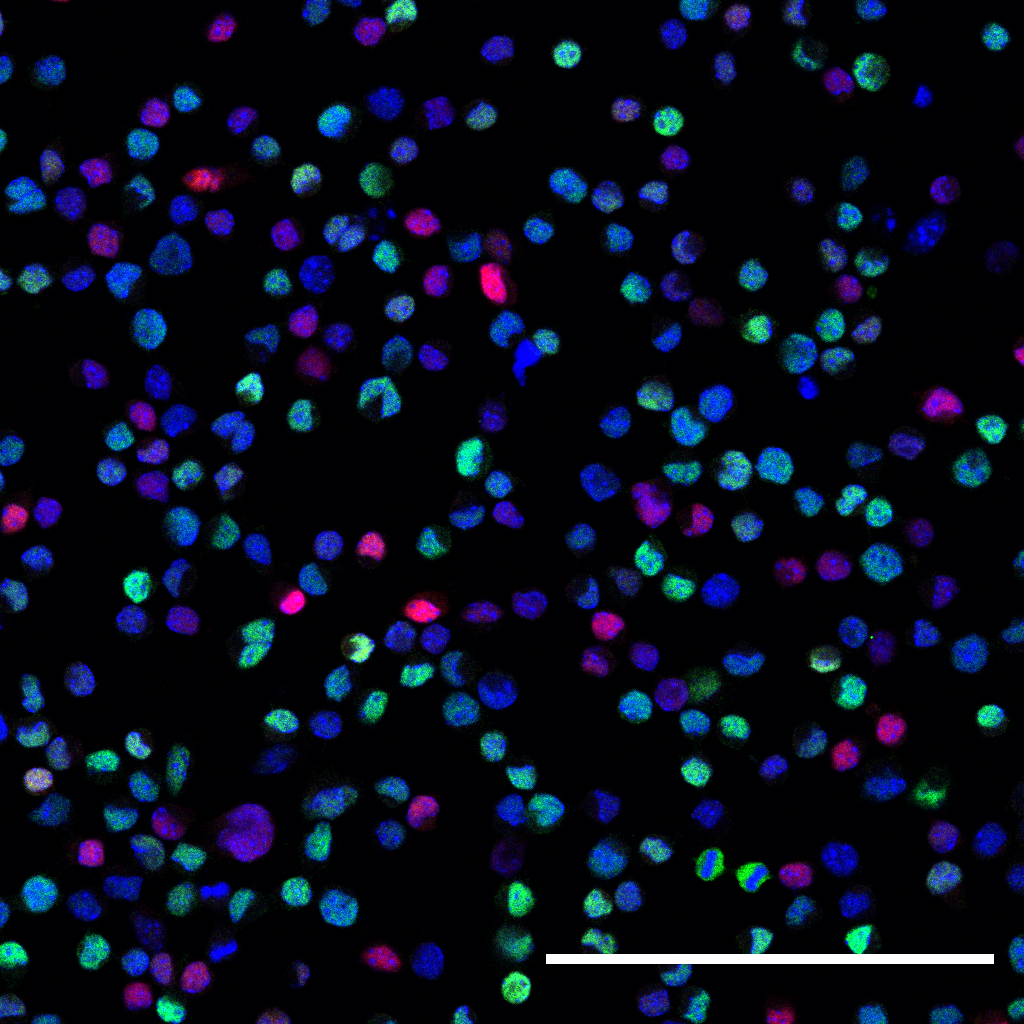

Supplement: Supplementary file 6 — Source data Fig. 4 [file 44319_2024_258_MOESM6_ESM.zip › Figure4/Fig4C/mGB1-KD-LY411575-merge.tif]

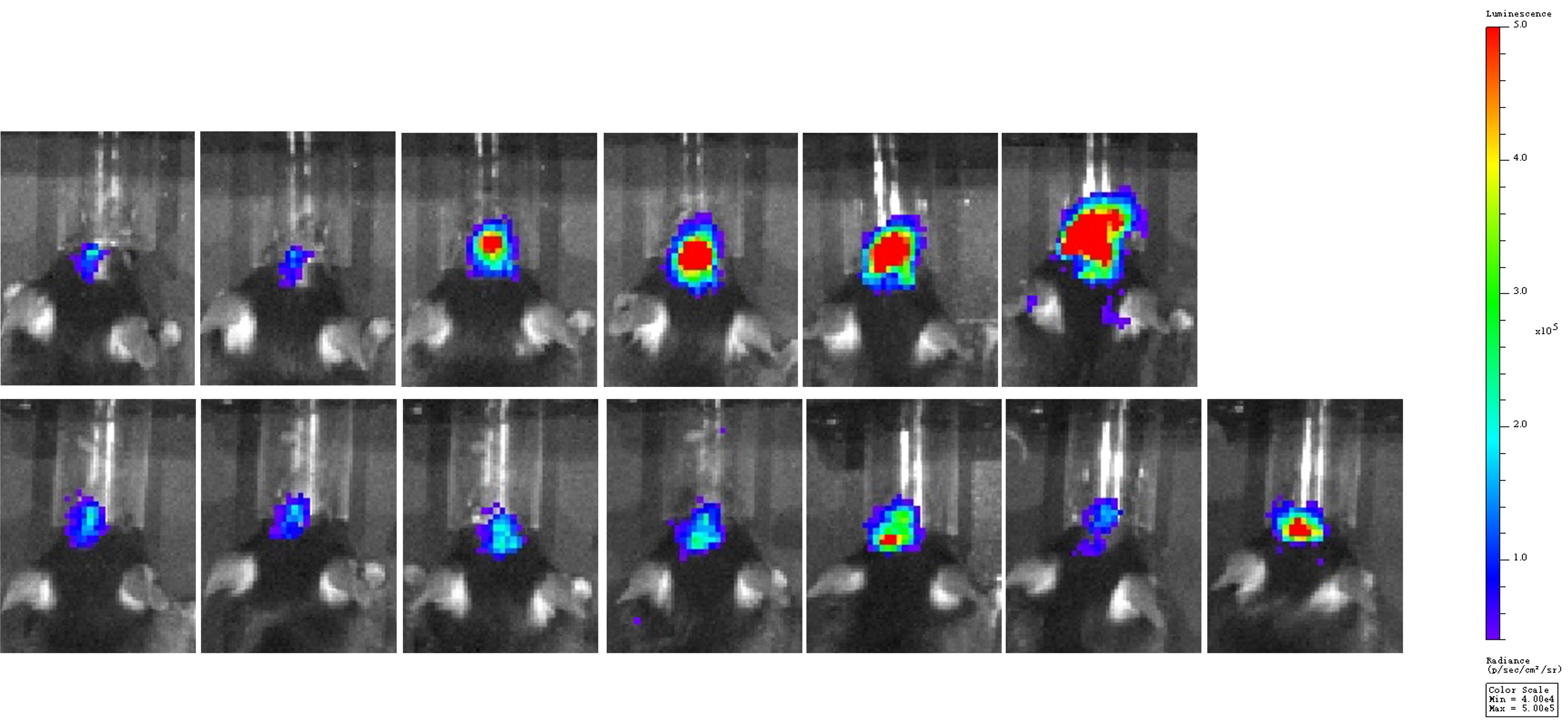

Supplement: Supplementary file 7 — Source data Fig. 5 [file 44319_2024_258_MOESM7_ESM.zip › Figure5/Fig5B/Figure5B_source_data.jpg]

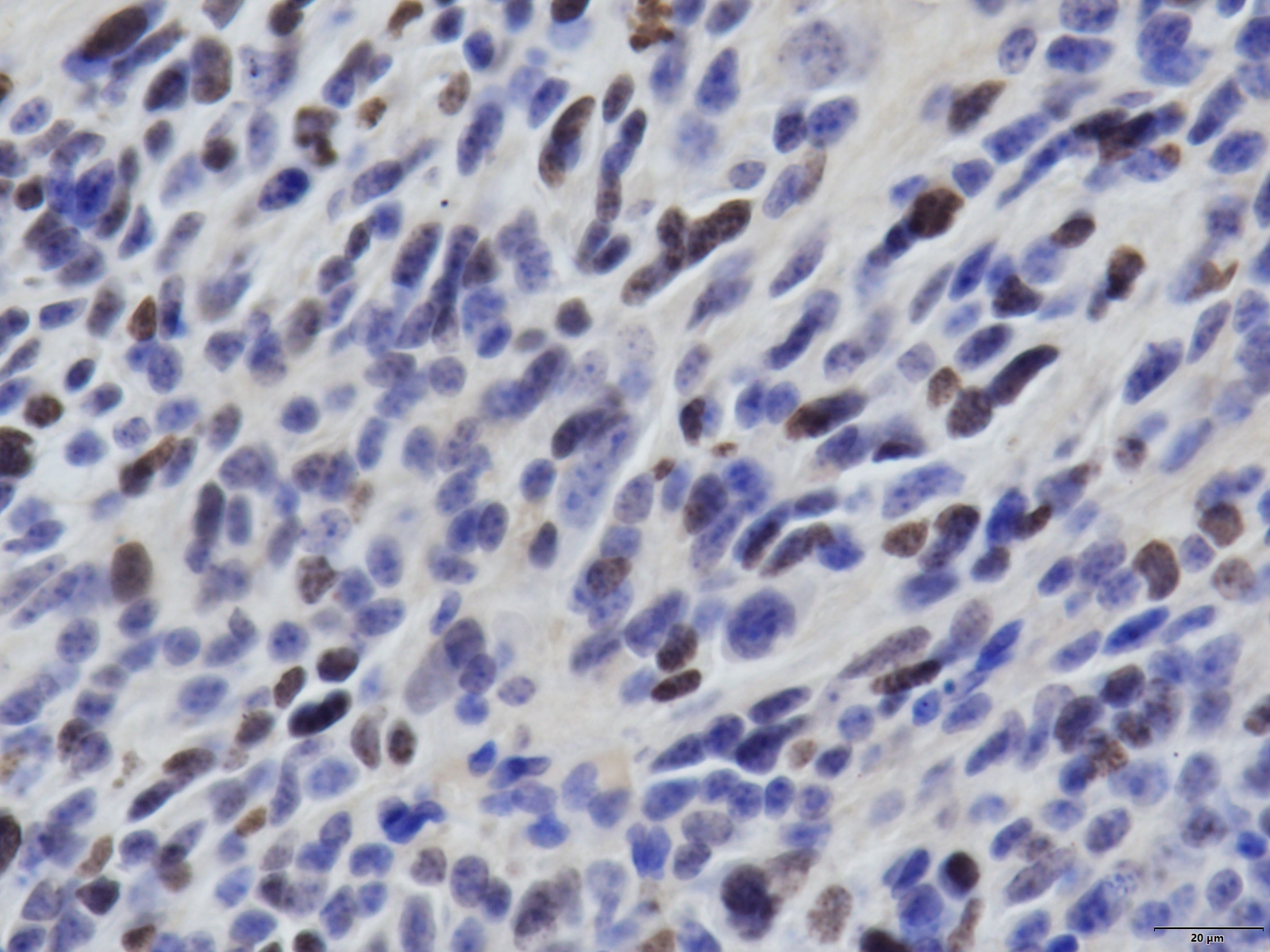

Supplement: Supplementary file 7 — Source data Fig. 5 [file 44319_2024_258_MOESM7_ESM.zip › Figure5/Fig5D/Combo_Ki67.jpg]

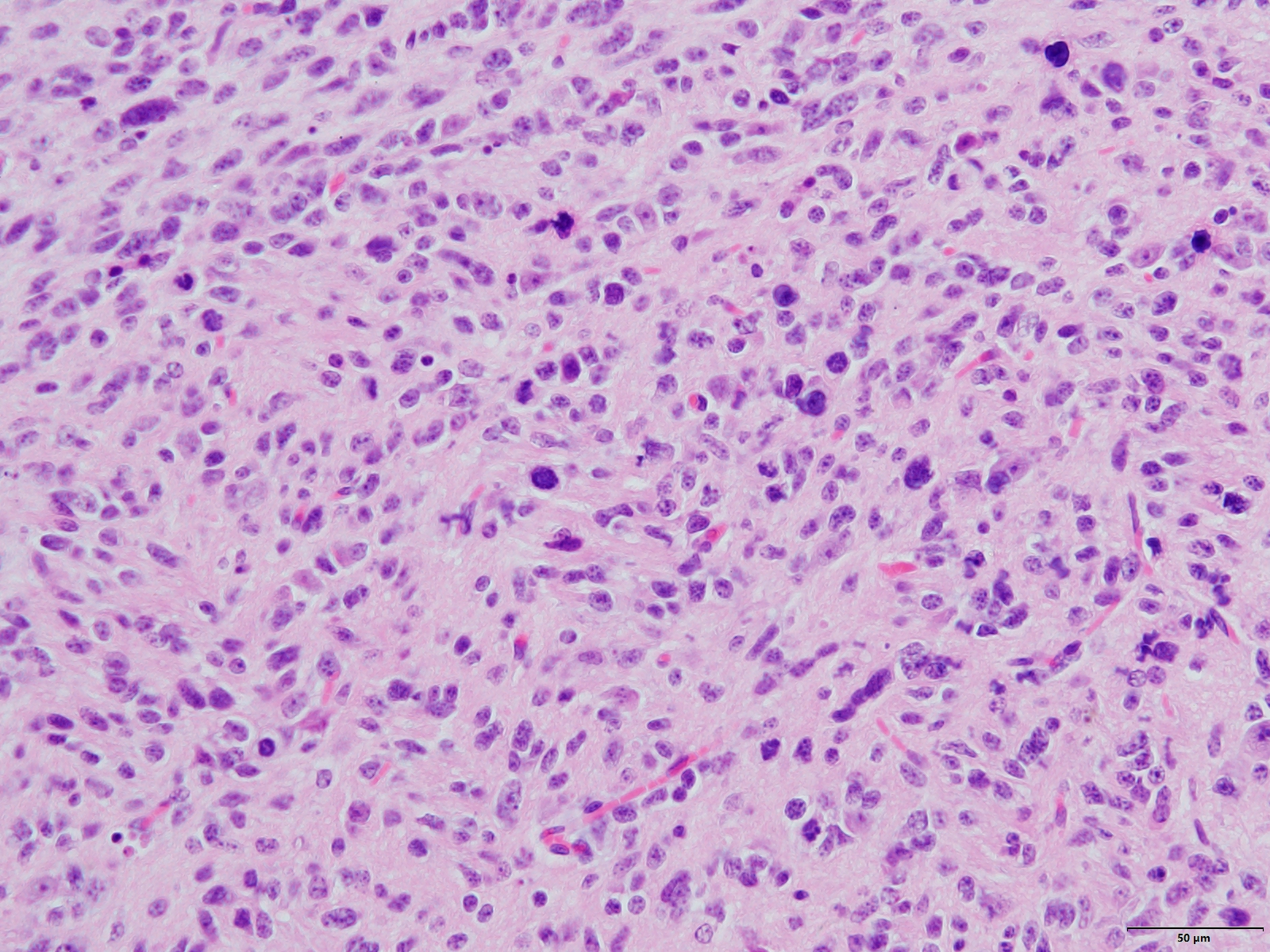

Supplement: Supplementary file 7 — Source data Fig. 5 [file 44319_2024_258_MOESM7_ESM.zip › Figure5/Fig5D/Como_HE.jpg]

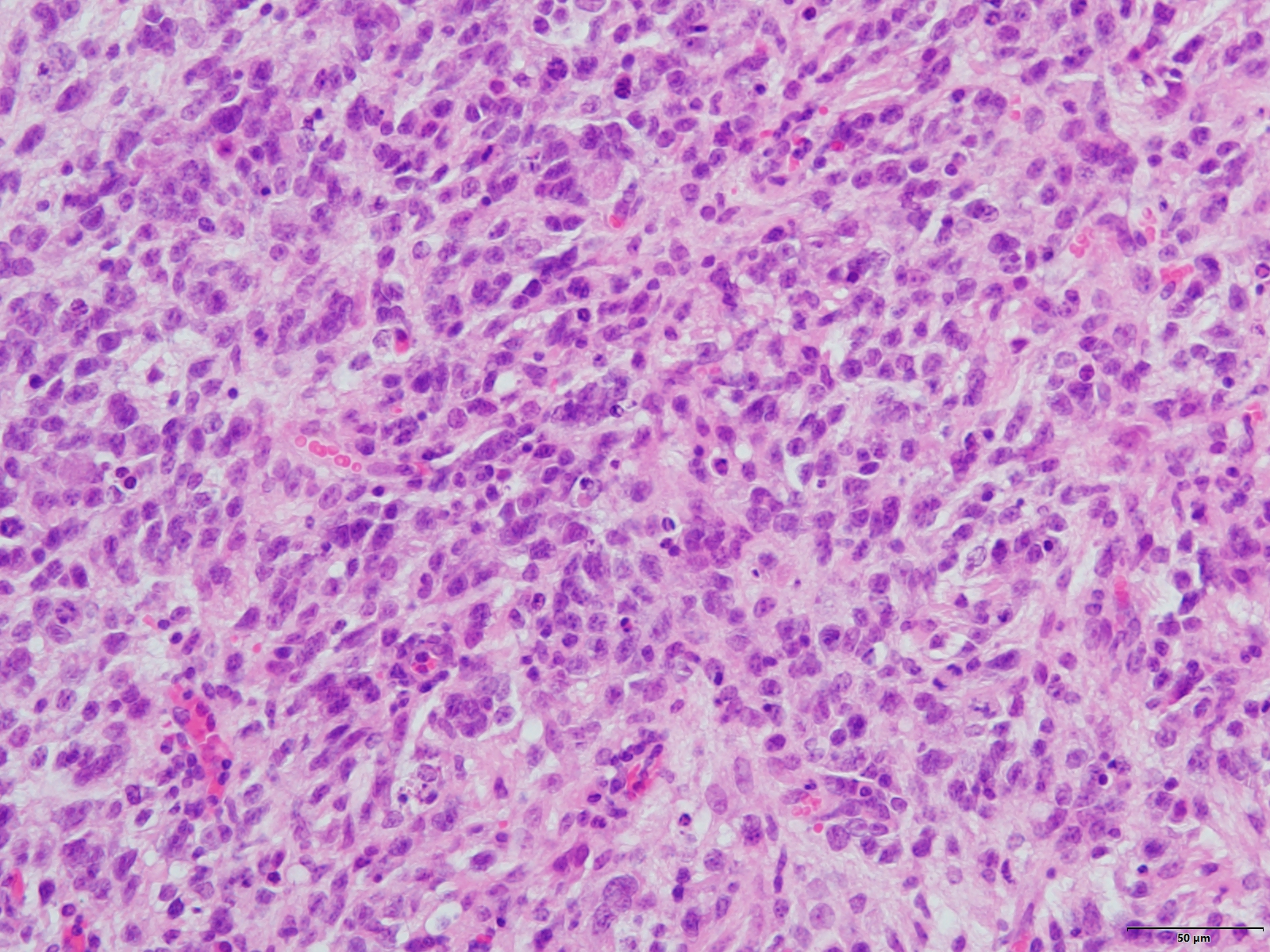

Supplement: Supplementary file 7 — Source data Fig. 5 [file 44319_2024_258_MOESM7_ESM.zip › Figure5/Fig5D/Vehicle_HE.jpg]

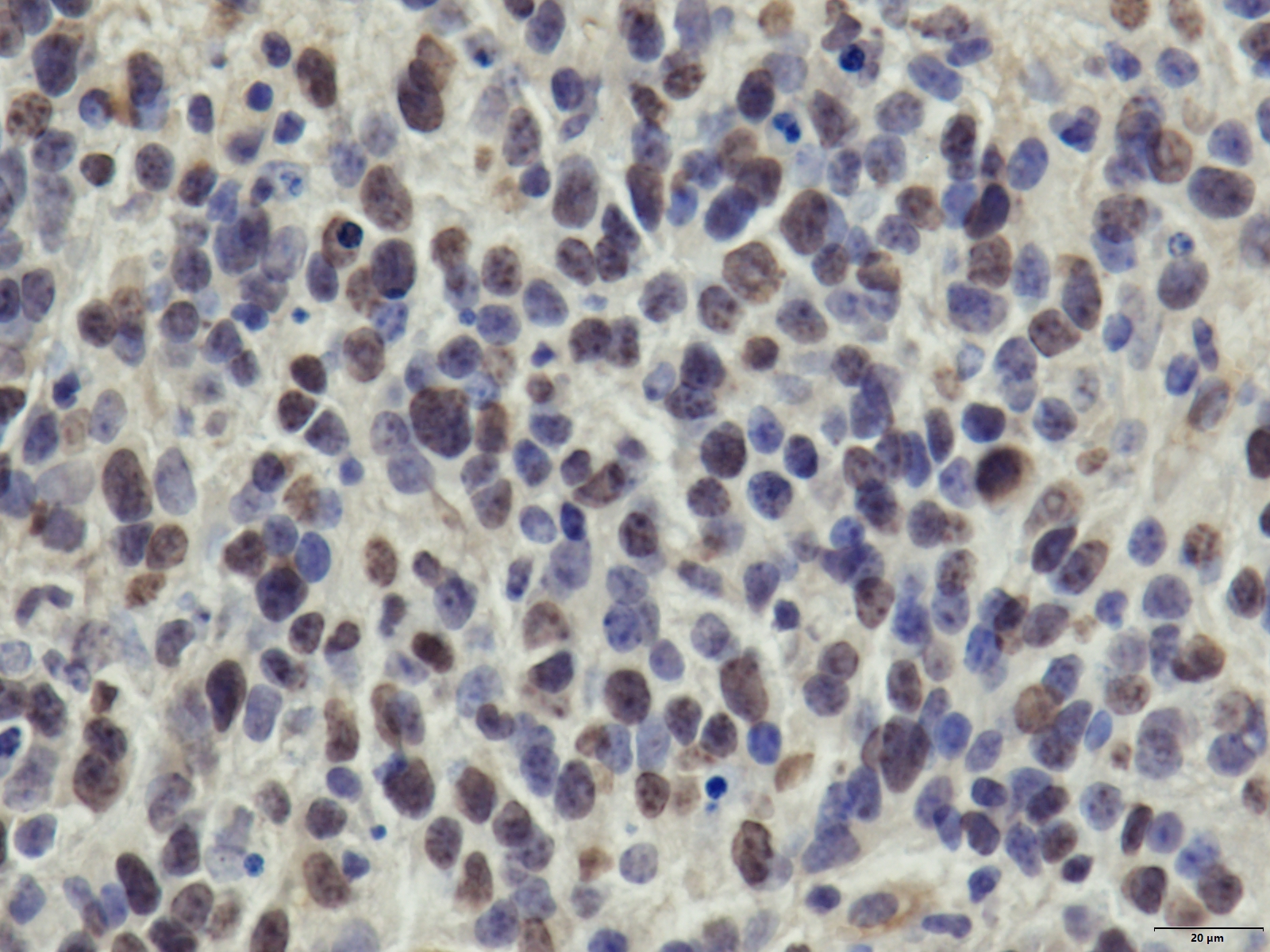

Supplement: Supplementary file 7 — Source data Fig. 5 [file 44319_2024_258_MOESM7_ESM.zip › Figure5/Fig5D/Vehicle_Ki67.jpg]
