# Supplementary material for: SOX10 mediates glioblastoma cell-state plasticity
Source: EMBO Rep. 2024 Sep 16;25(11):24. doi: 10.1038/s44319-024-00258-8 (PMC11549307; doi:10.1038/s44319-024-00258-8)
Supplement: Supplementary file 8 — Expanded View Figures [file 44319_2024_258_MOESM8_ESM.pdf]

Expanded View Figures

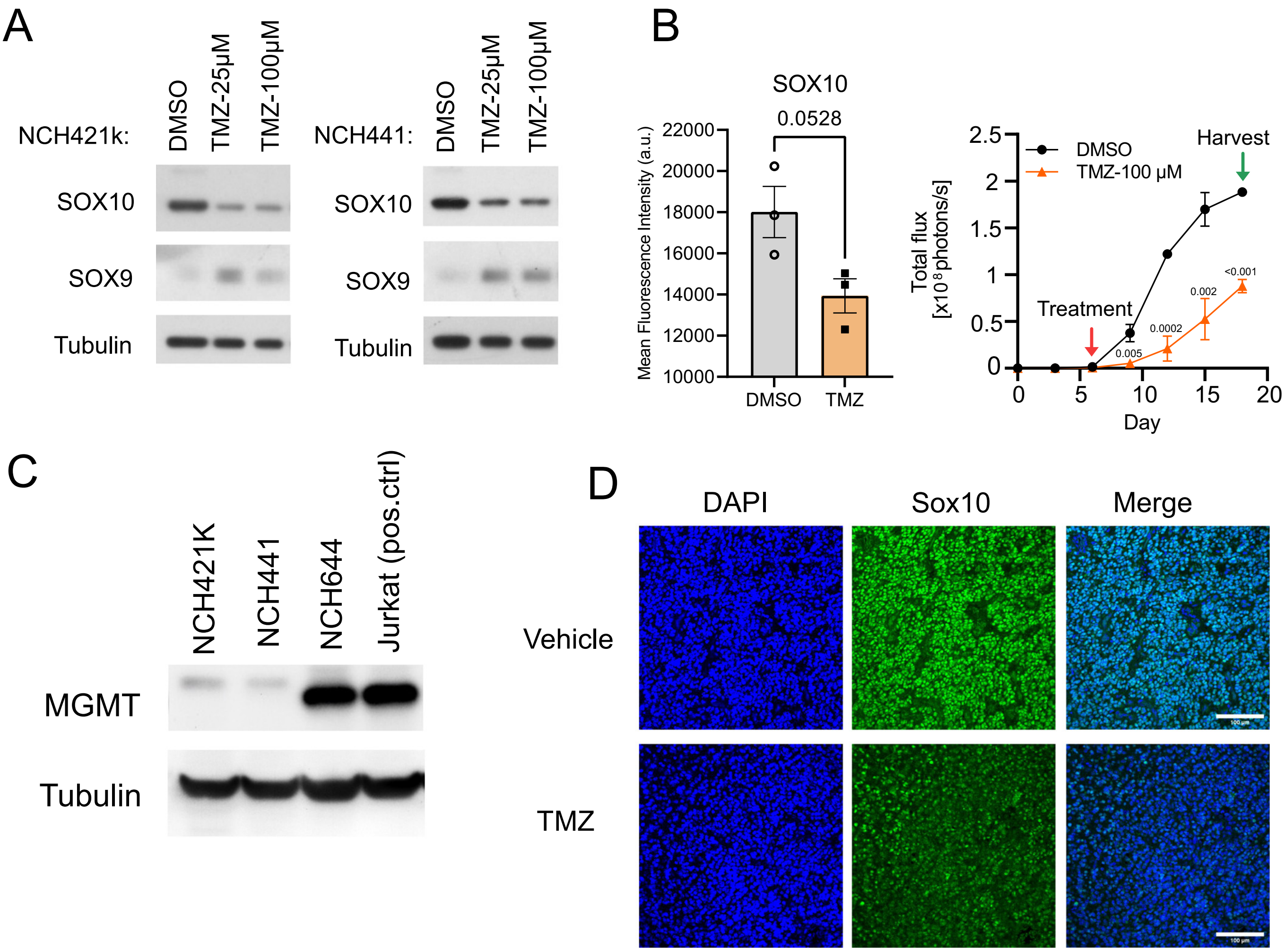

Figure EV1. Temozolomide represses SOX10 expression.

(**A**) Western blot analysis showing short-term (7-day) temozolomide treatment effects on SOX10 and SOX9 expression in NCH421k and NCH441 human glioblastoma cells. (**B**) Temozolomide-induced reduction of *SOX10* expression (left) and cell proliferation (right) of GFP and luciferase dual-labeled NCH644 glioblastoma cells growing in iPSC-derived cerebral organoids. Treatment with DMSO or 100 μM TMZ was started on day 6, and organoids were harvested on day 18. SOX10 expression was analyzed by recording mean fluorescence intensities by flow cytometry on day 18. Cell proliferation was monitored by bioluminescence (photon flux per second) imaging every 3 days. $n = 3$ organoids; mean ± SEM; P values were computed with two-tailed unpaired T tests. (**C**) Western blot analysis showing MGMT expression levels in SOX10-high glioblastoma stem cells. Protein lysate from Jurkat cells was used as the positive control for MGMT expression. (**D**) Immunofluorescence staining of Sox10 and Sox9 in the vehicle and TMZ-treated mice (Pdgfb/Akt RCAS glioblastoma mouse model) (Rusu et al, 2019). Scale bars = 100 μm. Related to Fig. 1.

© The Author(s)

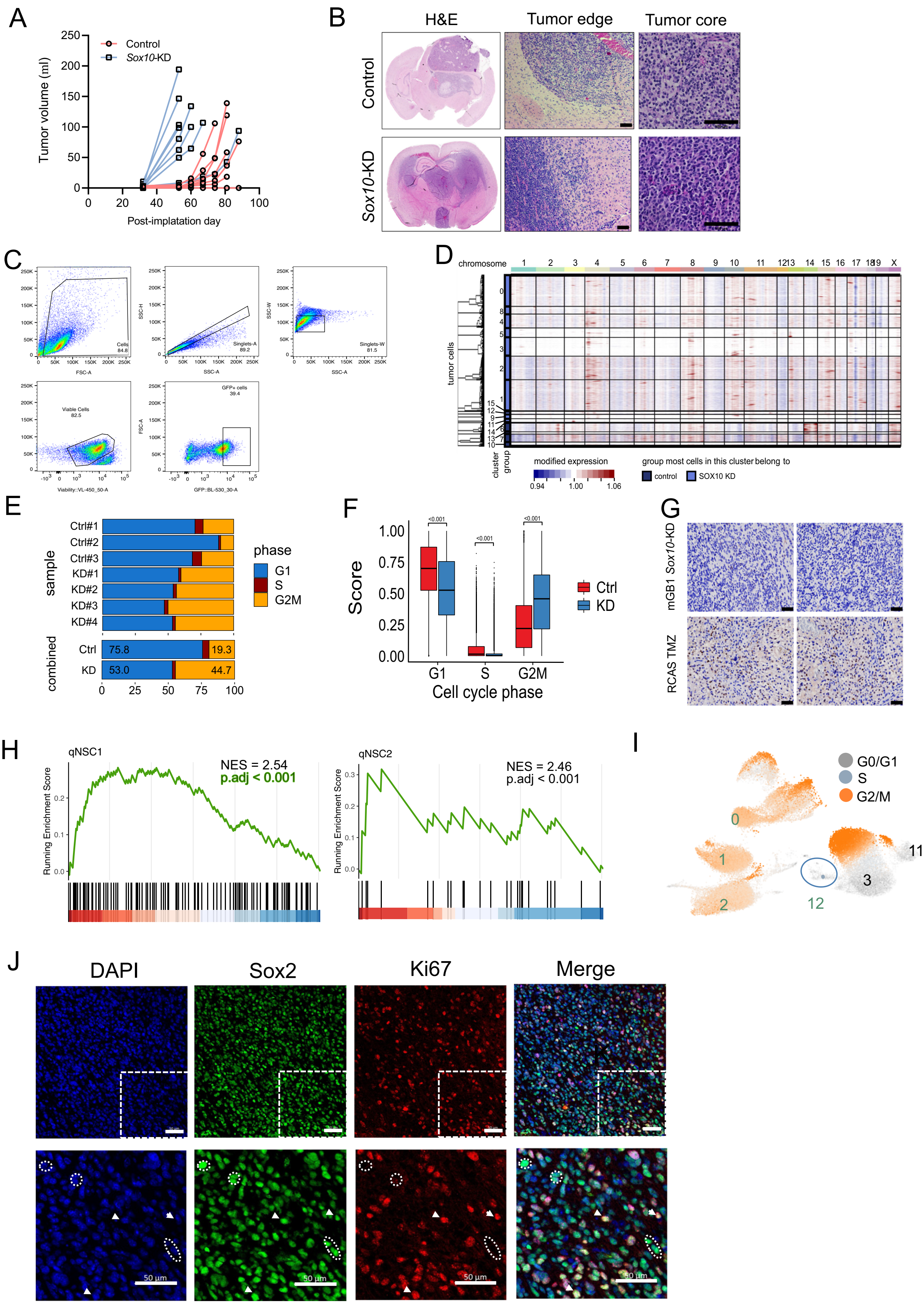
A
Control
Sox10-KD
Tumor volume (ml)
Post-implatation day
B
H&E
Tumor edge
Tumor core
Control
Sox10-KD
C
D
chromosome
tumor cells
modified expression
group most cells in this cluster belong to
control
SOX10 KD
E
Ctrl#1
Ctrl#2
Ctrl#3
KD#1
KD#2
KD#3
KD#4
sample
combined
phase
G1
S
G2M
75.8
19.3
53.0
44.7
F
Score
Cell cycle phase
Ctrl
KD
G
mGB1 Sox10-KD
RCAS TMZ
H
qNSC1
NES = 2.54
p.adj < 0.001
qNSC2
NES = 2.46
p.adj < 0.001
Running Enrichment Score
I
G0/G1
S
G2/M
J
DAPI
Sox2
Ki67
Merge
50 µm

 © The Author(s)

Figure EV2. Single-cell RNA sequencing analysis of control and Sox10-KD tumors.

(**A**) Tumor formation in C57BL6/J mice injected with mGB1 cells expressing non-targeting shRNA (control) and shRNA against Sox10 (*Sox10*-KD) (**B**) H&E staining of coronal brain sections of mice bearing Ctrl and KD tumors. Note that while both tumors resemble grade 4 glioblastoma, *Sox10*-KD tumors have a distinct bi-hemispheric growth pattern, as opposed to the bulky, circumscribed growth observed in the control tumors. Scale bar = 100 µm. (**C**) Gating strategies for isolating GFP-positive tumor cells. (**D**) Analysis of the copy number variations in GFP-positive tumor cells inferred by the R-package infercnv and using GFP-negative cells as reference. Tumor cells are arranged by Seurat clusters (rows) and chromosomes (columns). Copy number gains, red; losses, blue. (**E**) Cell cycle phase distributions of the individual tumors. (**F**) Boxplots showing G1, S and G2/M phase scores in control and *Sox10*-KD tumors. For G1, S and G2M phases, $n = 6747/31090$ cells, 293/1262 cells and 1296/25182 cells from Ctrl and KD tumors, respectively. The box-and-whisker plot shows the interquartile range (box), the median cell cycle scores (line inside the box), and the range of data within 1.5 times the interquartile range from the first and third quartiles (whiskers). *P* values were computed using two-tailed Wilcoxon's rank-sum tests. $^{***}P < 0.001$. (**G**) Immunohistochemistry staining of gamma H2AX (a DNA-damage marker) in KD tumors. TMZ-treated glioblastoma mouse model (bottom) is used as a positive control for the staining. Two fields of view were shown. Scale bars = 50 µm. (**H**) GSEA plot showing the enrichment of qNSC gene signatures (Kalamakis et al, 2019) in cluster 12. Adjusted *P* values (*P*.adj) were calculated using the Benjamini–Hochberg method. (**I**) UMAP visualization of the cell cycle phases of KD tumor cells. Cluster 12 is the qNSC-like cluster. Clusters 0, 1, 2 are the fast-cycling progenitor cell-like tumor cells. Cluster 3 and 11 are the more differentiated tumor cells. (**J**) Immunofluorescence images of co-staining Sox2 (a tumor stem cell marker) and Ki67 (a non-G0/proliferation marker) in KD tumors. The bottom right quadrant of the images were magnified in the bottom panel to highlight the presence of slow-cycling tumor stem cells (Sox2 + /Ki67-, dotted circles) and fast-cycling tumor stem cells (Sox2 + /Ki67 + , arrows). Scale bars = 50 µm. Related to Fig. 2.

© The Author(s)

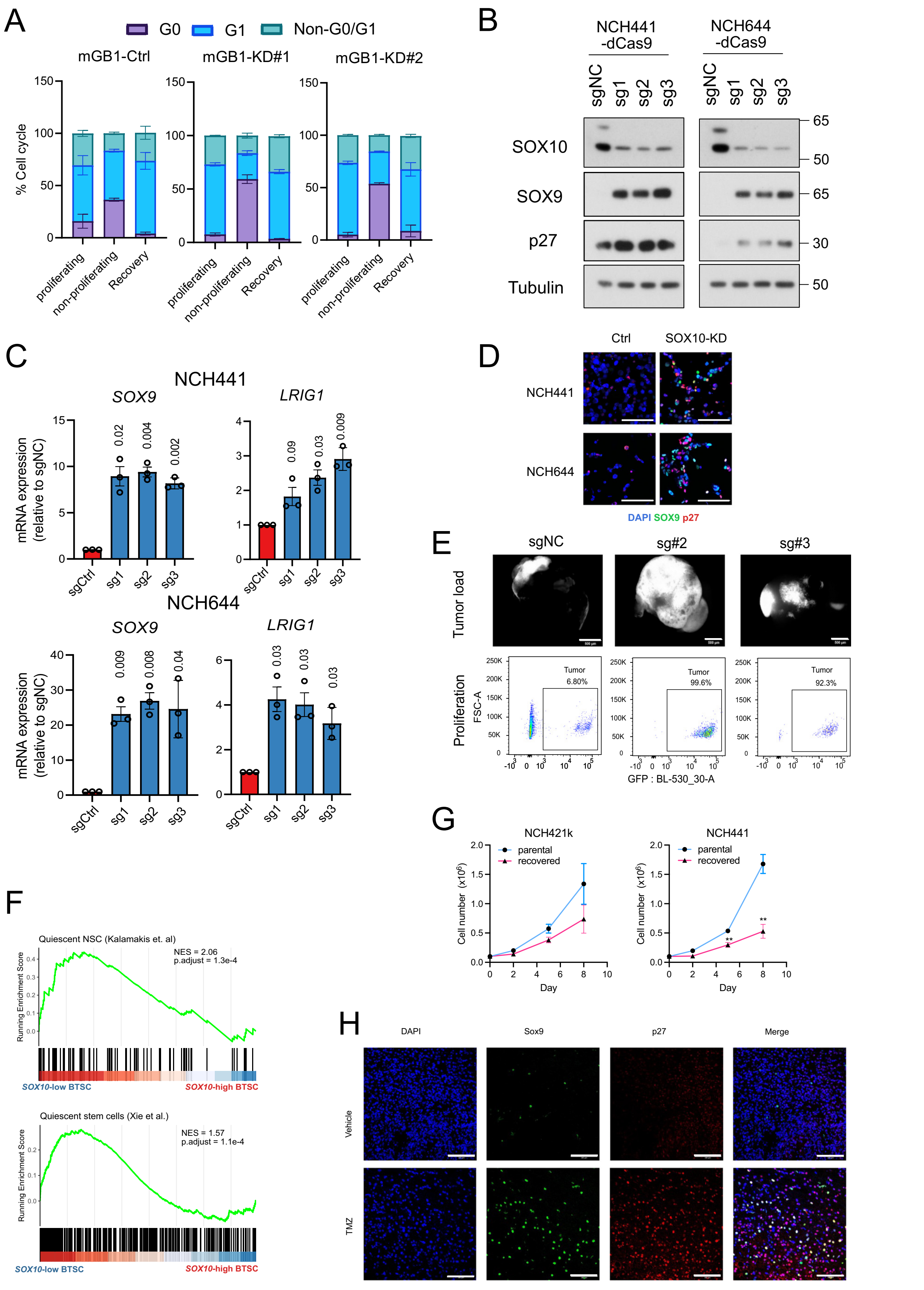
A
G0 G1 Non-G0/G1
mGB1-Ctrl
mGB1-KD#1
mGB1-KD#2
% Cell cycle
proliferating
non-proliferating
Recovery
B
NCH441 -dCas9
NCH644 -dCas9
sgNC sg1 sg2 sg3
SOX10
SOX9
p27
Tubulin
C
NCH441
SOX9
LRIG1
mRNA expression (relative to sgNC)
sgCtrl sg1 sg2 sg3
NCH644
D
Ctrl
SOX10-KD
NCH441
NCH644
DAPI SOX9 p27
E
sgNC
sg#2
sg#3
Tumor load
Proliferation
FSC-A
Tumor
GFP : BL-530_30-A
F
Quiescent NSC (Kalamakis et. al)
NES = 2.06
p.adjust = 1.3e-4
Running Enrichment Score
SOX10-low BTSC
SOX10-high BTSC
Quiescent stem cells (Xie et al.)
NES = 1.57
p.adjust = 1.1e-4
G
NCH421k
NCH441
parental
recovered
Cell number (x10^6)
Day
H
DAPI
Sox9
p27
Merge
Vehicle
TMZ

Figure EV3. SOX10 suppression induces quiescent stem cell features.

(**A**) Bar plots showing the distribution of mGB1 control and *Sox10*-KD cells in the G0, G1 and non-G0/G1 cell cycle phases. Error bars represent mean ± SEM from three independent experiments. (**B**) Western blot showing the upregulation of SOX9 and p27 in control cells (sgNC) and *Sox10*-KD cells (transduced with sg1-3) in SOX10-high NCH441 and NCH644 cells. The molecular weights (in KDa) of the detected proteins are indicated on the right. (**C**) RNA expression of the qNSC markers *SOX9* and *LRIG1*. Mean values ± SEM relative to mGB1 control cells are shown; $N = 3$ biological replicates; P values were calculated with one-sample *T* test. (**D**) Immunofluorescence images of NCH441 and NCH644 control and *SOX10*-KD cells co-stained with *SOX9* and p27. Scale bars = 100 µm. (**E**) GFP-tagged NCH644 control (sgNC) and *SOX10*-KD (sg#2 and sg#3) co-cultured with iPSC-derived cerebral organoids. Stereoscopic fluorescence images showing the growth on day 14 after co-culture. Scale bar = 500 µm. The scatter plots below show the quantification of the tumor load by GFP flow cytometry. (**F**) GSEA plots showing the enrichment of slow-cycling stem cell signatures in SOX10-low vs. SOX10-high BTSCs. Adjusted *P* values (*P*.adj) were calculated using the Benjamini–Hochberg method. (**G**) Growth curves of NCH421k and NCH441 parental cells and cells that have recovered after TMZ treatment. Mean values ± SEM from three experiments are shown. P values were calculated using a two-tailed unpaired *t* test. (**H**) Sox9 and p27 immunofluorescence staining of tumor sections from mice treated with DMSO or TMZ (100 mg/kg, for 5 consecutive days). Scale bars = 100 µm. Related to Fig. 3.

© The Author(s)

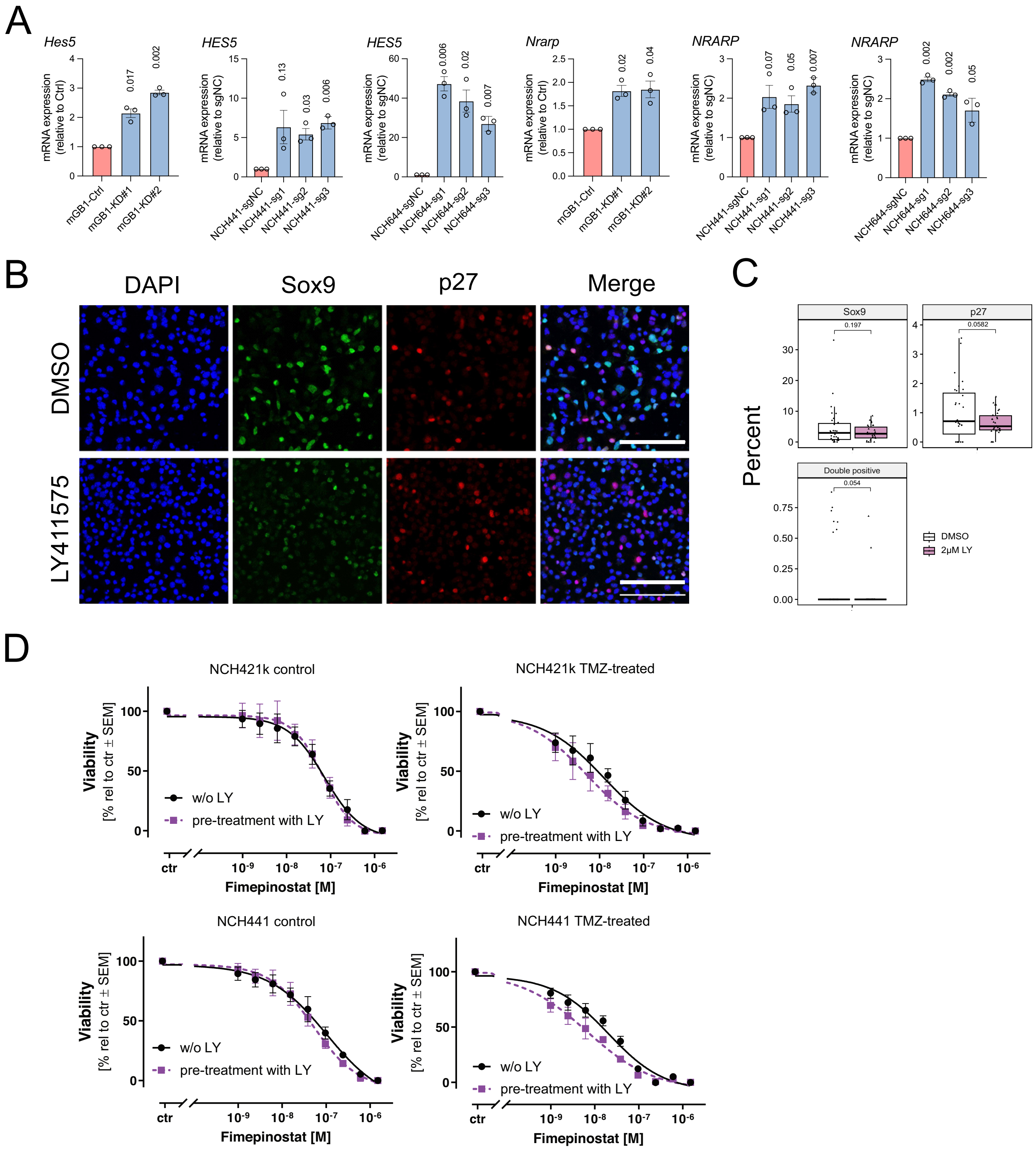
A
Hes5
HES5
HES5
Nrarp
NRARP
NRARP
mRNA expression (relative to Ctrl)
mRNA expression (relative to sgNC)
0.017
0.002
0.13
0.03
0.006
0.006
0.02
0.007
0.02
0.04
0.07
0.05
0.007
0.002
0.002
0.05
mGB1-Ctrl
mGB1-KD#1
mGB1-KD#2
NCH441-sgNC
NCH441-sg1
NCH441-sg2
NCH441-sg3
NCH644-sgNC
NCH644-sg1
NCH644-sg2
NCH644-sg3
B
DAPI
Sox9
p27
Merge
DMSO
LY411575
C
Percent
Sox9
0.197
p27
0.0582
Double positive
0.054
DMSO
2μM LY
D
NCH421k control
NCH421k TMZ-treated
NCH441 control
NCH441 TMZ-treated
Viability
[% rel to ctr ± SEM]
w/o LY
pre-treatment with LY
ctr
Fimepinostat [M]

© The Author(s)

Figure EV4. Notch pathway upregulation and inhibition in Sox10-KD cells.

(**A**) Quantification of relative RNA expression of Notch target genes (*HES5* and *NRARP*) in mouse and human glioblastoma spheroid cultures. Data are represented as mean ± SEM expression levels relative to mGB1 control cells. $n = 3$ biological replicates. *P* values were calculated using one-sample T tests. (**B**) Immunofluorescence images of mGB1 control cells stained with Sox9 and p27. Cells were cultured in reduced growth factor conditions with and without LY411575 treatment. Scale bars = 100 µm. (**C**) Boxplots showing the results of the quantifications of the percentages of Sox9-positive, p27-positive and Sox9 and p27 double-positive cells. Each dot represents the percentage of cells from an image ($n = 30$ images for each condition). The box-and-whisker plot shows the interquartile range (box), the median values (line inside the box), and the range of data within 1.5 times the interquartile range from the first and third quartiles (whiskers). *P* values were computed using two-tailed unpaired T tests. (**D**) Dose-response curves of Fimepinostat alone (black curve) and LY411575 + Fimepinostat (purple curve) in human glioblastoma cells (NCH421k and NCH441) with and without TMZ pre-treatment. Shown are the mean cell viability (%) ± SEM from three technical replicates of one experiment. Related to Fig. 4.

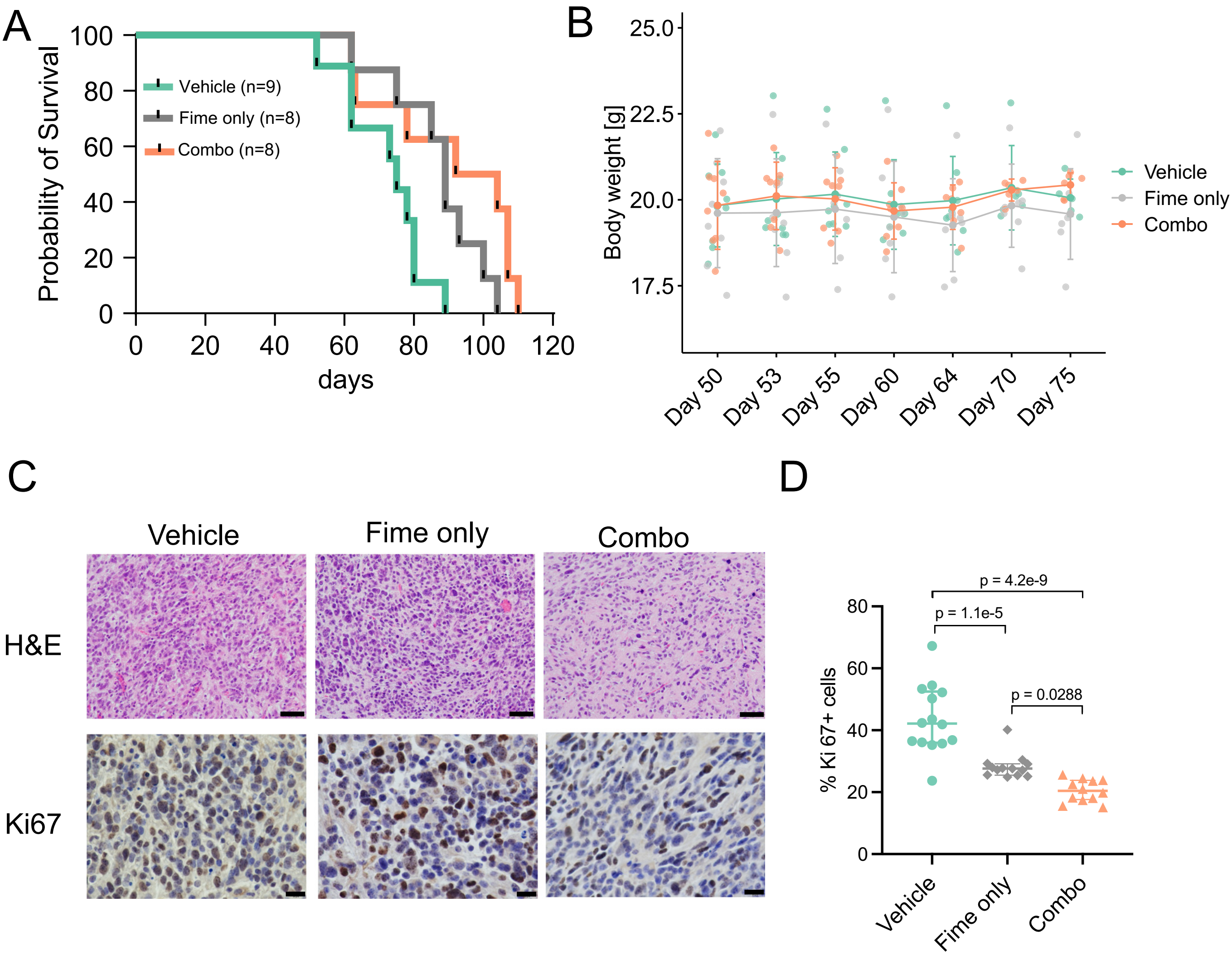

Figure EV5. Fimepinostat treatment of mGB1 tumors in vivo.

(**A**) Kaplan–Meier survival curves of mice intracranially injected with KD cells in Vehicle ($n = 9$), Fime only ($n = 8$) and Combo groups ($n = 8$), respectively. (**B**) Body weight of animals prior to randomization (Day 50) and during the course of the treatment (Day 53 to Day 75). On day 50, the vehicle group had $n = 9$ animals, the Fime only group had $n = 8$ animals and the Combo group had $n = 8$ animals. Shown are the mean values ± SD. (**C**) H&E (top) and Ki67 staining (bottom) of end-stage tumors of each treatment group. Images of the Vehicle and Combo groups were reused from Fig. 5D. Scale bars for top and bottom panels are 50 µm and 20 µm, respectively. (**D**) Quantification of Ki67 positive cells in (**C**). Shown are the median values ± interquartile ranges. *P* values were calculated with one-way ANOVA with Tukey post-hoc tests to test the level of significance among treatment groups ($n = 12$–13 images from three different animals in each group). Related to Fig. 5.

 © The Author(s)
